# Estimated Deaths Averted in Adults by COVID-19 Vaccination in Select Latin American and Caribbean Countries

**DOI:** 10.1093/ofid/ofae528

**Published:** 2024-09-10

**Authors:** Alexandra Savinkina, Daniel M Weinberger, Cristiana M Toscano, Lucia H De Oliveira

**Affiliations:** Department of Epidemiology of Microbial Diseases and Public Health Modeling Unit, Yale School of Public Health, Yale University, New Haven, Connecticut, USA; Department of Epidemiology of Microbial Diseases and Public Health Modeling Unit, Yale School of Public Health, Yale University, New Haven, Connecticut, USA; Institute of Tropical Pathology and Public Health, Federal University of Goias, Goiania, GO, Brazil; Independent Consultant, working at Comprehensive Immunization Program, Pan-American Health Organization (PAHO) when the project was conceived

**Keywords:** Caribbean, COVID-19, Latin America, mathematical modeling, vaccination

## Abstract

**Background:**

The coronavirus disease 2019 (COVID-19) pandemic has had a significant impact on global health, with millions of lives lost worldwide. Vaccination has emerged as a crucial strategy in mitigating the impact of the disease. This study aims to estimate the number of deaths averted through vaccination in Latin America and the Caribbean region (LAC) during the first year and a half of vaccination rollout (January 2021–May 2022).

**Methods:**

Publicly available data on COVID-19 deaths and vaccination rates were used to estimate the total number of deaths averted via vaccination in LAC. Using estimates for number of deaths, number of vaccinated, and vaccine effectiveness, a counterfactual estimated number of deaths observed without vaccination was calculated. Vaccine effectiveness estimates were obtained from published studies. The analysis focused on 17 countries in LAC and considered adults aged 18 years and older.

**Results:**

After accounting for underreporting, the analysis estimated that >1.49 million deaths were caused by COVID-19 in the selected countries during the study period. Without vaccination, the model estimated that between 2.10 and 4.11 million COVID-19 deaths would have occurred. Consequently, vaccination efforts resulted in ∼610 000 to 2.61 million deaths averted.

**Conclusions:**

This study represents the first large-scale, multicenter estimate of population-level vaccine impact on COVID-19 mortality in LAC. The findings underscore the substantial impact of timely and widespread vaccination in averting COVID-19 deaths. These results provide crucial support for vaccination programs aimed at combating epidemic infectious diseases in the region and future pandemics.

The World Health Organization (WHO) declared a public health emergency of international concern on January 30, 2020, in response to the identification of a novel coronavirus (SARS-CoV-2) in China. In March 2020, as laboratory-confirmed SARS-CoV-2 cases exponentially grew in China and newly identified transmission was reported in other countries, the WHO declared the outbreak a global pandemic and called on countries to rapidly respond with control and mitigation plans to slow the spread of the virus. In the ensuing months, countries worldwide faced challenges to keep a responsive pace with the spread of the virus, which led to substantial health and socioeconomic losses and significant mortality related to coronavirus disease 2019 (COVID-19) worldwide [1]. A total of >7 million deaths due to COVID-19 have been reported globally since January 1, 2020.

Countries in the Americas have been among the hardest hit by the pandemic. By early 2023, ∼43% of all reported COVID-19 deaths in the world had occurred in the region, reaching 2.89 million deaths as of January 1, 2023. As of spring 2023, South America as a subregion experienced 1.35 million COVID-19 deaths during the pandemic [2]. Although most COVID-19 deaths occur among older adults and individuals living with comorbidities, deaths have occurred in all ages including young children, even though younger ages appear to be less susceptible to severe disease.

The first vaccines to prevent COVID-19 became available for use in late 2020 in the United States and Europe and in early 2021 in Latin American and the Caribbean (LAC). As of July 2021, 8 COVID-19 vaccines had received Emergency Use Listing (EUL) by the WHO prequalification process [3] upon meeting predefined criteria for safety and efficacy. By May 2023, this number had reached 15 [4], and many more were under assessment for prequalification [5].

The rapid deployment of vaccines has been proven critical to halt the pandemic's toll in the region. Many countries in the region accessed vaccines via PAHO's Revolving Fund, in active collaboration with the COVAX Facility. In LAC countries, 82% of the population has received at least 1 dose of COVID-19 vaccine as of spring 2023, and 1.1 billion doses of vaccine have been administered [6]. Despite the initial availability of COVID-19 vaccines across the region, wide inter- and intracountry variation in access to and availability of vaccines has been observed in the region [6].

Selected studies have recently used models to estimate the impact of COVID-19 vaccination in averting COVID-19 deaths in selected countries and periods [7, 8]. To date, the burden of COVID-19 deaths prevented by vaccination, a key measure of the impact of the vaccination program, has not been established for the LAC region. This measure is important to policy-makers as it can inform future decision-making on the effectiveness and efficiency of large-scale vaccination campaigns, and it can provide a data point for future studies evaluating COVID-19 response in the region. This study aims to estimate the numbers of COVID-19 deaths averted due to vaccination in selected countries of the LAC region during the COVID-19 pandemic.

## METHODS

### Study Design, Setting, and Period

**Figure 1.**
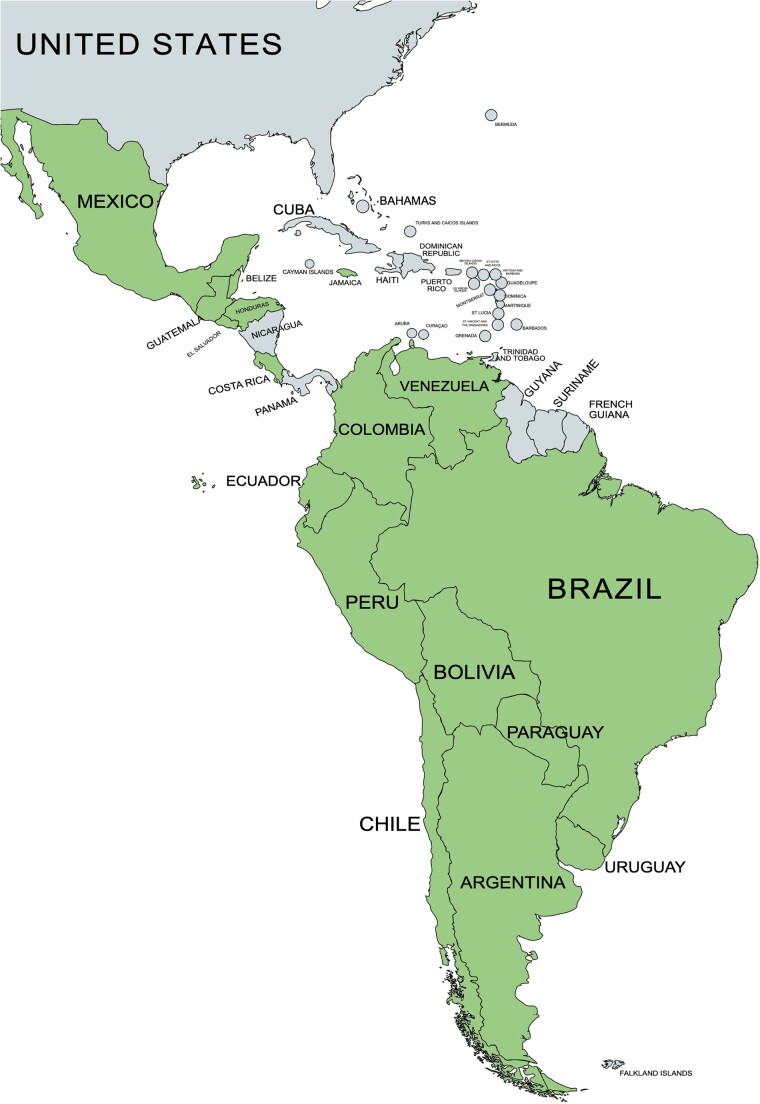
Map showing 17 countries in the Latin American and Caribbean regions that were included in the modeling analysis. Included countries are shown in gray and excluded countries in green. Included countries are Argentina, Brazil, Chile, Colombia, Paraguay, Uruguay, Jamaica, Peru, Belize, Bolivia, Costa Rica, Ecuador, El Salvador, Guatemala, Honduras, Mexico, and Venezuela.

We used existing data on reported deaths due to COVID-19 and COVID-19 vaccination coverage over time to estimate deaths averted via vaccination for select countries in Latin America and the Caribbean. This modeling study used data from 17 countries in the LAC region during the period ranging from vaccine introduction in each country (early 2021) to May 2022 (Table 1). These 17 countries were selected for inclusion because they had available COVID-19 vaccination coverage data (Figure 1). They included Argentina, Belize, Bolivia, Brazil, Chile, Colombia, Costa Rica, Ecuador, El Salvador, Guatemala, Honduras, Jamaica, Mexico, Paraguay, Peru, Uruguay, and Venezuela. All other countries in the Latin American and Caribbean regions were excluded due to missing data on COVID-19 vaccination coverage and/or COVID-19 deaths. Our analysis considered only adults over 18, and the model was stratified by age (18–59 years old, 60 years and older).

**Table 1.** Country-Specific Data on Vaccination, Including Start Date of Vaccination, Percent Coverage by Age Groups Considered in the Analysis, and Vaccines Used During Period

Country	Start Date of Vaccination	% Age 60+ Years Vaccinated by May 2022	% Age 18–59 Years Vaccinated by May 2022	Vaccines in Use Through May 2022^a^
Argentina	2020–12	95	90.8	CanSino, Moderna, Oxford/AstraZeneca, Pfizer/BioNTech, Sinopharm/Beijing, Sputnik V
Brazil	2021–01	98.4	85.9	Johnson&Johnson, Pfizer/BioNTech, Oxford/AstraZeneca, Sinovac
Chile	2020–12	93.9	96.9	CanSino, Moderna, Oxford/AstraZeneca, Pfizer/BioNTech, Sinovac
Colombia	2021–03	81.1	48.3	Johnson&Johnson, Moderna, Oxford/AstraZeneca, Pfizer/BioNTech, Sinovac
Paraguay	2021–02	76	59.9	Covaxin, Moderna, Oxford/AstraZeneca, Pfizer/BioNTech, Sinopharm/Beijing, Sinovac, Sputnik V
Uruguay	2021–03	100	79.8	Sinovac, Pfizer/BioNTech, Oxford/AstraZeneca
Jamaica	2021–03	35.2	26.4	Johnson&Johnson, Moderna, Pfizer/BioNTech, Oxford/AstraZeneca
Peru	2021–02	90.2	91.1	Moderna, Oxford/AstraZeneca, Pfizer/BioNTech, Sinopharm/Beijing
Belize	2021–02	74.7	67.8	Johnson&Johnson, Oxford/AstraZeneca, Pfizer/BioNTech, Sinopharm/Beijing
Bolivia	2021–02	70.9	62.6	Johnson&Johnson, Oxford/AstraZeneca, Pfizer/BioNTech, Sinopharm/Beijing, Sputnik V
Costa Rica	2021–02	85.1	90.4	Oxford/AstraZeneca, Pfizer/BioNTech
Ecuador	2021–02	90.5	87.1	CanSino, Oxford/AstraZeneca, Pfizer/BioNTech, Sinovac
El Salvador	2021–02	84.2	80.8	Oxford/AstraZeneca, Pfizer/BioNTech, Sinopharm/Beijing, Sinovac
Guatemala	2021–02	58.8	47.6	Moderna, Oxford/AstraZeneca, Pfizer/BioNTech, Sputnik V
Honduras	2021–02	66.4	34.6	Johnson&Johnson, Moderna, Oxford/AstraZeneca, Pfizer/BioNTech, Sputnik V
Venezuela	2021–02	42.2	58	Abdala, Sinopharm/Beijing, Sinovac, Soberana02, Sputnik Light, Sputnik V
Mexico	2020–12	92.7	80.1	CanSino, Johnson&Johnson, Moderna, Oxford/AstraZeneca, Pfizer/BioNTech, Sinovac, Sputnik V

Abbreviation: COVID-19, coronavirus disease 2019.

^a^Source: Our World in Data COVID-19 vaccine database [9].

### Data Sources

#### COVID-19 Deaths

When possible, we used country-specific data for observed deaths over time from the COVerAGE-DB global demographic database of COVID-19 deaths [10]. This data set used data as reported by government entities, such as health ministries, and harmonized the data to standard metrics, measures, and age bands using the penalized composite link model for ungrouping [11]. The COVerAGE-DB database provided deaths by age group (18–59, 60+) as well as by time period (collapsed into monthly death counts). COVerAGE-DB was used for observed death counts in Argentina, Brazil, Chile, Colombia, Paraguay, Uruguay, Jamaica, and Peru. Mexico had COVID-19 mortality data available by age and time in COVerAGE-DB through October 2021, and these data were used for the analysis from December 2020 to October 2021. For countries that did not have COVID-19 death data reported in COVerAGE-DB either for the full time period of the analysis or for a part of it, we used non-age-stratified COVID-19 death estimates over time from the World Health Organization Coronavirus (COVID-19) Dashboard [1]. Data from the WHO were not age-stratified; therefore, we imputed age-stratified deaths using a linear regression model. The outcome variables in this regression were the proportion of deaths in each country and the month and year in which death occurred among individuals over age 60 years. Covariates included population proportion under 60 and vaccination proportion in those 60 and over (part and full), as well as a categorical variable for month/year. The linear regression model was validated using data from 8 countries for which age-stratified death data were available for the entire time period. Additional methods and validation results can be found in the Supplementary Data. Countries for which death data were imputed from WHO-reported numbers for the full time period of the analysis were Belize, Bolivia, Costa Rica, Ecuador, El Salvador, Guatemala, Honduras, and Venezuela. In addition, age-specific death counts were imputed for Mexico from November 2021 to May 2022. Due to the imputation required for age-specific vaccination in Mexico (described in the “Vaccine Coverage and Effectiveness Data” section), Mexico was not included in the linear regression model to impute age-specific deaths.

To account for the underreporting of COVID-19 deaths in the region, we used a multiplier considering available evidence from the literature, considering country-specific mortality underestimates ranging from 0% to 79% in the studied countries (see “Additional Methods”) [12].

#### Vaccine Coverage and Effectiveness Data

Data on vaccination by age over time by country and age group were obtained from the Pan-American Organization (PAHO) [13] and from Our World in Data (OWID) [9]. Vaccine coverage considered partial and complete primary series and first booster dose (not the second booster and beyond). OWID data were complete for all countries of interest but were not age-stratified and included vaccination in those under 18, which was outside our study population. Data reported to PAHO were age-stratified, available as of September 2021, and less complete for some countries. Therefore, we used PAHO data to determine the proportion of all vaccinations that were administered to each age group and OWID to determine the proportion of the population within each age group that was vaccinated over time. Data for age-specific vaccination were only available for Mexico starting in February of 2022. We therefore assumed the same proportion of vaccines going to each age group in the months from December 2020 through January 2022 as in February 2022. As it is likely that elderly people were more prioritized for early vaccination, it is likely that this led us to slightly underestimate [14–16] the number of deaths averted in the older age group in the early part of 2021.

Multiple vaccine products were available and used by countries within PAHO over the course of vaccine administration (Table 1), and it is difficult to ascertain what proportion of all vaccinations were using specific products at each time point. Therefore, we considered a range of vaccine effectiveness against death measures (stratified by partial vs full vs booster vaccination) based on the vaccine effectiveness against death estimated for the vaccine products in the literature, with an effort to use studies conducted in Latin American countries [17–26]. Additional boosters beyond the first were not considered in the analysis. Estimates of vaccine effectiveness against death can be seen in Table 2.

**Table 2.** Parameters Used as Inputs to the Model

	Estimate	Source
COVID-19 mortality underreporting rate	Country dependent (see “Additional Methods”)	Msemburi et al. [12]
Vaccine effectiveness against death estimates, by age and dose		
Medium vaccine effectiveness, 18–59 y	% (point estimate)	
Fully vaccinated (2-dose primary schedule)	0.849	Santos et al. [20]Cerqueira-Silva et al. [22]
Partly vaccinated (1-dose primary schedule)	0.553	Cerqueira-Silva et al. [21],Santos et al. [20]
1st booster dose	0.971	Cerqueira-Silva et al. [22]Ranzani et al [23]Jara et al [26]Menni et al. [25]
Medium vaccine effectiveness, 60+ y		
Fully vaccinated (2-dose primary schedule)	0.801	Monteiro et al. [14]Santos et al. [20]Ranzani et al. [24]Hitchings et al. [15]
Partly vaccinated (1-dose primary schedule)	0.526	Bermimgham et al. [16]Nunes et al. [19]
1st booster dose	0.925	Cerqueira-Silva et al. [22]Ranzani et al. [23]Jara et al [26]Menni et al. [25]
Vaccine effectiveness against death estimates, sensitivity analyses, by age and dose		
High vaccine effectiveness, 18–59 y		
Fully vaccinated (2-dose primary schedule)	0.945	Santos et al. [20]
Partly vaccinated (1-dose primary schedule)	0.625	Cerqueira-Silva et al. [21]Santos et al. [20]
1st booster dose	0.99	Cerqueira-Silva et al. [22]Ranzani et al. [23]Jara et al [26]Menni et al. [25]
High vaccine effectiveness, 60+ y		
Fully vaccinated (2-dose primary schedule)	0.936	Hitchings et al. [15]
Partly vaccinated (1-dose primary schedule)	0.737	Bermimgham et al. [16]Nunes et al. [19]
1st booster dose	0.99	Cerqueira-Silva et al. [22]Ranzani et al. [23]Jara et al [26]Menni et al. [25]
Low vaccine effectiveness, 18–59 y		
Fully vaccinated (2-dose primary schedule)	0.678	Cerqueira-Silva et al. [22]
Partly vaccinated (1-dose primary schedule)	0.353	Cerqueira-Silva et al. [21]Santos et al. [20]
1st booster dose	0.90	Cerqueira-Silva et al. [22]Ranzani et al. [23]Jara et al. [26]Menni et al. [25]
Low vaccine effectiveness, 60+ y		
Fully vaccinated (2-dose primary schedule)	0.612	Ranzani et al. [23]
Partly vaccinated (1-dose primary schedule)	0.157	Bermimgham et al. [16]Nunes et al. [19]
1st booster dose	0.90	Cerqueira-Silva et al. [22]Ranzani et al. [23]Jara et al [26]Menni et al. [25]

Abbreviation: COVID-19, coronavirus disease 2019.

#### Population

Population estimates by age subgroup for each country in 2021 were obtained from the United Nations World Populations Prospects [27].

### Model Structure and Equations

To calculate deaths averted via vaccination, we used the following equations, wherein is the number of $D_{i,m}$ deaths reported in month i for age group m. The reported level of vaccination for age group m at month i is shown as ***v***. $D_{i,m,V=0}$ is the expected number of deaths that would have been observed for age group m at month i had there been no vaccination, and **X** is a multiplier for assumed underreporting of case counts [18]. $p_{o,i,m}$ is the proportion of the population partially vaccinated (1 dose), $p_{f,i,m}$ is the proportion of the population fully vaccinated, and $p_{b,i,m}$ is the proportion of the population booster vaccinated for age group m at month i. $VE_{o,}$ is the vaccine effectiveness against death for partial vaccination, $VE_{f}$ is the vaccine effectiveness against death for full series vaccination, and $VE_{b}$ is the vaccine effectiveness against death for full series vaccination with booster. $D_{Averted\;}$ is the estimated number of deaths averted via vaccination.

Equation 1 shows the calculation for expected deaths that would have been observed if there had been no vaccination in age group m in month i.


(1) $$D_{i,m,V=0}=\frac{D_{i,m,V=v}*X}{1-(p_{o,i,m}*VE_{p})-(p_{f,i,m}*VE_{f})-(p_{b,i,m}*VE_{b})}$$

Equation 2 sums all months of observation for age group m.


(2) $$D_{V=0,m}=\sum_{1}^{i}D_{V=0,i,m}$$

Equation 3 calculates estimated deaths averted via vaccination.


(3) $$D_{Averted,m\;}=\;D_{V=0,m}-D_{V=v,m}$$

Equation 4 sums deaths averted across age groups for the final estimate of deaths averted.


(4) $$D_{Averted\;}=\sum_{1}^{length(m)}D_{Averted,m}$$

We calculated estimated deaths averted by country within the group of countries included in the regional analysis and used country-specific estimates to come to a final region-wide estimate.

### Sensitivity Analysis

As there were multiple vaccine products used in this region over time, with varying estimates of vaccine effectiveness against COVID-19 due to different variants of concern (VOC), which also vary over time and by country, we considered a range of vaccine effectiveness estimates in the model, assigned to low, medium, and high range estimates. Also, in a sensitivity analysis, we modeled estimates with and without accounting for underreporting of COVID-19 mortality.

### Ethical Issues

This study used publicly available secondary data, considering a variety of open data sources and platforms that report COVID-19-related data made available by the countries, PAHO, WHO, and other international organizations. Only aggregated data were used, and mathematical models have been applied to estimate the averted death burden associated with COVID-19 vaccination in the region.

## RESULTS

**Figure 2.**
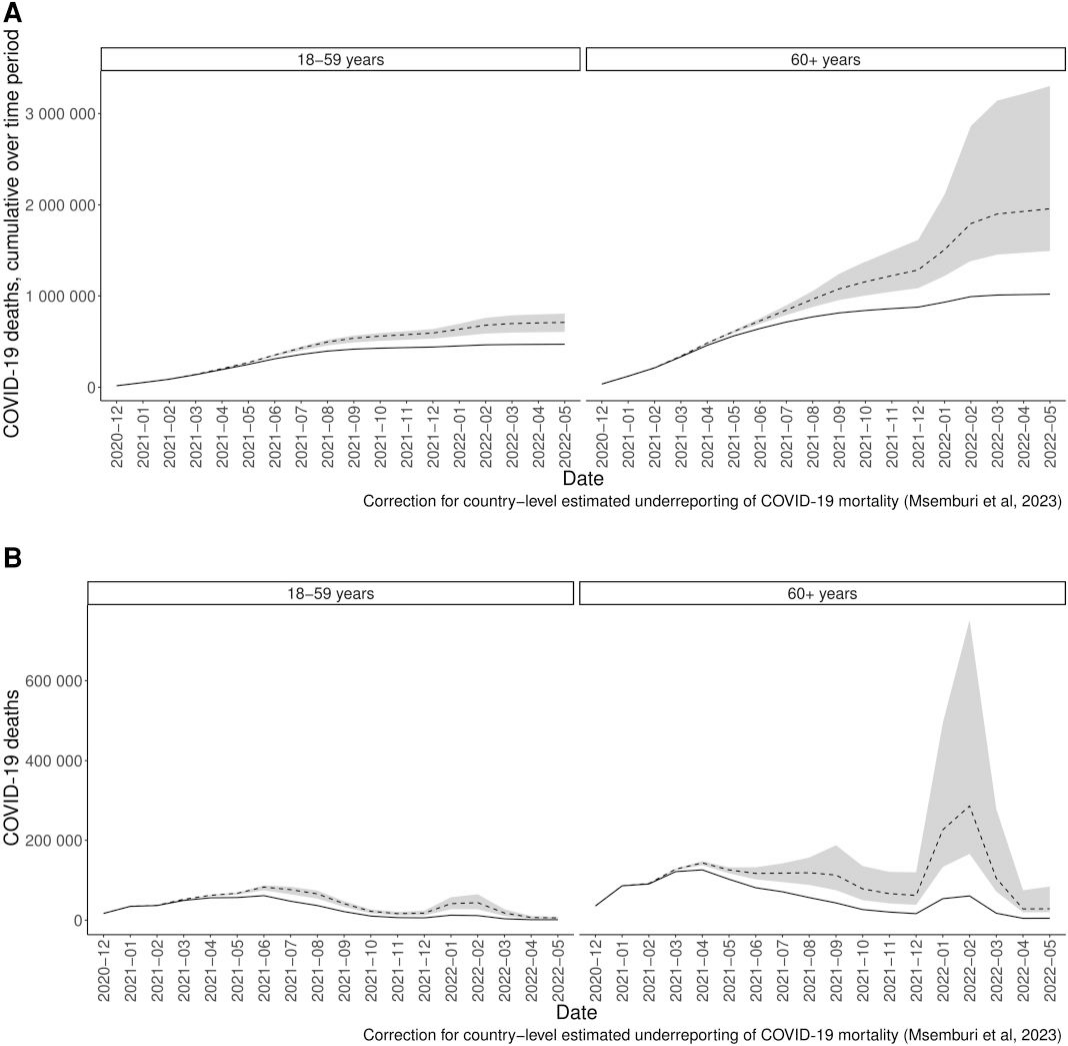
Observed deaths (solid line) and model estimates for deaths without vaccination (dashed line) by age group (60+: blue; 18–59: red) over time. *A*, Cumulative deaths over time. *B*, Incident deaths over time. The shaded gray area represents the range of deaths averted given the range of vaccine effectiveness estimates. Abbreviation: COVID-19, coronavirus disease 2019.

There were 1.05 million COVID-19 deaths reported in the 17 selected LAC countries from the start of vaccination (ranging by country from December 2020 to March 2021) to May 2022. Accounting for underreporting, the analysis assumes that there were likely 1.49 million COVID-19 deaths in these countries during this period (Table 3, Figure 2). Our model estimates that without vaccination and assuming medium vaccine effectiveness, there would have been 2.67 million deaths during this same time period. Therefore, an estimated 1.18 million deaths were averted by vaccination, with that estimate ranging between 610 000 deaths averted assuming low vaccine effectiveness and 2.62 million deaths averted assuming high vaccine effectiveness. Overall, our model estimates that ∼273 (142–607) deaths were averted per 100 000 people in LAC.

**Table 3.** Model Estimates for Deaths Averted by COVID-19 Vaccination, Stratified by Vaccine Effectiveness Ranges, With and Without Correction for Underreporting of Deaths During the Study Period; Selected Countries in the Latin American and Caribbean Region

		Medium Vaccine Effectiveness		Low Vaccine Effectiveness		High Vaccine Effectiveness	
	Observed Deaths	Estimated No. of Deaths, No Vaccination	Estimated Deaths Averted	Estimated No. of Deaths, No Vaccination	Estimated Deaths Averted	Estimated No. of Deaths, No Vaccination	Estimated Deaths Averted
Region estimates							
Correction for underreporting	1 490 000	2 670 000	1 180 000	2 100 000	610 000	4 110 000	2 620 000
No correction for underreporting	1 050 000	1 920 000	870 000	1 500 000	450 000	3 010 000	1 960 000

Abbreviation: COVID-19, coronavirus disease 2019.

In a sensitivity analysis, if we instead considered COVID-19 deaths as reported with no assumed underreporting, our model estimated that vaccination averted 870 000 deaths (range, 450 000–1.96 million) (Table 3; Supplementary Figure 1).

Country-specific data closely align with region-wide results (Supplementary Table 1, Supplementary Figures 2 and 3). Rates of deaths averted by country varied considerably by country over time, especially in the 60 and over age group (Supplementary Figure 4).

## DISCUSSION

The COVID-19 pandemic has caused massive loss of life worldwide, and vaccination has emerged as a critical tool to mitigate its impact. To our knowledge, this is the first multicountry study evaluating the impact of vaccination on COVID-19 deaths in the Latin American and Caribbean region. Our model estimates that between 610 000 and 2.6 million deaths were averted by vaccination, underscoring the importance that vaccination had in mitigating the impact of the COVID-19 pandemic. Large-scale vaccination in a pandemic setting was difficult, and countries in the LAC region faced various challenges when implementing these campaigns. Our study results can help inform policy- and decision-makers of the outcome of these campaigns. This will be useful in decision-making in future epidemic scenarios where a vaccine is available.

The incidence of deaths averted by country varied based on vaccination coverage over time (especially in the 60 and over age group) (Supplementary Figure 4) as well as whether COVID-19 outbreaks were seen largely before or after the initiation of vaccination. For instance, the model estimates that proportionally more deaths were averted in Chile and Uruguay as there were spikes in deaths at a time when a lot of the population (especially those 60 and over) was vaccinated (Supplementary Figures 2 and 3).

Our results are in line with available model projections of COVID-19 deaths averted by vaccination in other regions and in selected countries. The projected impact of COVID-19 vaccination on deaths during the first year of the COVID-19 vaccination rollout globally during the first year of vaccine availability from December 8, 2020, through December 8, 2021, was reported to be 14.4 million (95% credible interval [Crl], 13.7–15.9) in 185 countries [28].

Savinkina et al. [8] also modeled COVID-19 deaths averted by vaccinating low-income and lower- to middle-income countries in late 2021, when the Omicron VOC was already predominant globally. The study estimated that scaling up global vaccination in 2022 with complete primary series plus a booster dose could result in an additional 1.5 million COVID-19 deaths averted in low- and lower- to middle-income countries.

Country- and region-specific studies have also been conducted. In the United States, Steele et al. [7] modeled the projected number of deaths averted by COVID-19 vaccination and concluded that the US vaccination campaign averted 58% of deaths that might have otherwise occurred in the period of December 1, 2020, through September 30, 2021. Over the same time period, our analysis estimates that ∼25% (range based on vaccine effectiveness estimates, 15%–34%) of deaths were averted via vaccination in LAC. When expanding the time frame through May 2022, our analysis estimates that ∼45% (range based on vaccine effectiveness estimates, 30%–65%) of deaths were averted via vaccination. This may be due to vaccination starting earlier and being more readily and equitably accessible to the population in the United States than in much of LAC.

Ferreira et al. [29] estimated averted COVID-19 deaths by vaccination in Brazil among adults aged 60 years and older considering a time period from January to August 2021, reporting 58 000 averted deaths. The authors reinforce that this represents an underestimate of the impact of vaccination in the Brazilian elderly. In this time period, our model estimates ∼70 000 deaths averted in those aged 60 years and older (range, 47 000–90 000).

A similar assessment for 33 countries of the European region from December 2020, when vaccination was first introduced, through November 2021 resulted in an estimated 469 186 deaths averted (95% Crl, 129 851–733 744; 23%–62%) [30].

Despite varying geographical locations and scope and different study periods, all these studies reinforce the significant impact of averted COVID-19 mortality due to vaccination, with varying rates by country, period, and vaccine coverage.

Our study has several limitations. A number of vaccine products were used in the countries in the analysis over the time period of interest, but we did not have adequate data on vaccine coverage by each vaccine product. Therefore, we averaged vaccine effectiveness across products and present 3 estimates, ranging from low vaccine effectiveness equal to the lowest-effectiveness vaccine used in the region to high vaccine effectiveness, equal to the effectiveness of mRNA vaccines. Due to uncertainty in vaccine effectiveness, we did not consider vaccine waning in the analysis as we provide a wide range of vaccine effectiveness estimates that would capture vaccine waning. Our analysis relies on publicly available aggregated data as reported by countries, and some of this reporting may be incomplete due to the nature of data reporting during a pandemic. Some of this limitation, particularly related to underreporting of COVID-19 mortality, was addressed by considering an adjustment factor for underreporting. In addition, our analysis does not account for protection derived from past SARS-CoV-2 natural infection against reinfection and severe disease [31]. This could affect our estimates of vaccine impact on deaths in our case-base scenario. This is somewhat accounted for in the sensitivity analysis considering a range of estimates for potential vaccine effect. We did not consider life-years gained in addition to deaths averted by vaccination as an outcome; as COVID-19 most severely impacted the elderly, the results of such an analysis may look somewhat different and could be considered in future work. Lastly, we did not use a dynamic transmission model and therefore cannot model disease dynamics in the population over time or the indirect protection resulting from vaccination (herd immunity). Considering the reported indirect effects of the vaccine in decreasing the probability of secondary transmission from an infected vaccine recipient to another individual [32], potentially resulting in an even more significant impact of the vaccine in terms of reducing deaths, our estimates are conservative and likely underestimate vaccine impact. Due to all of these uncertainties, the range for deaths averted by vaccination presented in this analysis is very wide. However, we believe the estimate to still be useful to decision-makers as it gives an idea of the magnitude of vaccine effect. Country-specific data can be found in the Supplementary Data, which may provide a more granular idea of vaccine effect within nations.

This analysis captured data from 17 countries for which data were available. Though there are more countries in Latin America and the Caribbean, the countries excluded from the analysis were mainly small and would not greatly change the magnitude of the overall estimated results. However, these countries are largely low income and had a high burden of COVID-19 disease; it is important to continue working to improve data collection and surveillance in this region in order to make analyses possible in the future. Also, our model does not take into account other public health measures implemented by the countries during the pandemic or adherence to them by the population, including mask use, lockdowns, and quality and availability of health care services, among others. Though these factors may impact COVID-19 mortality, it was not possible to ascertain their individual impact in this modeling study.

Finally, it is worthwhile to mention that such an analysis is made possible by the availability of administrative data collected and shared by countries. During a pandemic and in public health emergency situations, data collection, cleaning, sharing, and analysis may be even more challenging and pose an additional burden to already constrained public health care personnel at the local level. However, analyses such as this one underscore the importance of data collection and analysis to support decision-making, public health policy implementation, and assessment of interventions.

Future directions of this work include more in-depth analyses of countries with additional available data in order to assess differences in vaccine effectiveness by specific region or population group (including socioeconomic status) and evaluation of potential alternative vaccination scenarios. Though such analyses were beyond the scope of this analysis due to data limitations, this work would be valuable in further informing policy-makers when considering vaccination policy.

In conclusion, our study provides evidence of the significant impact of vaccination in reducing COVID-19 deaths in the Latin American and Caribbean region, one of the regions most strongly impacted by the pandemic. Despite the many challenges to COVID-19 vaccination in LAC—including timely access to vaccines, varying vaccine products and schedules, evolving circulating variants, and shifting vaccination strategies and target groups—these findings underscore the substantial impact of timely and widespread vaccination in averting COVID-19 deaths. Further studies evaluating the impact of vaccination in other selected health outcomes including hospitalization, health care service utilization, costs, and long-COVID, in addition to other nonhealth outcomes including educational, social, and economic indicators, may provide additional evidence relevant to policy-makers and society as a whole. All these constitute complementary pieces of evidence that are valuable support for vaccination programs aimed at combating epidemic infectious diseases in the region and future pandemics.

## Supplementary Material

ofae528_Supplementary_Data

## Supplementary Data

Supplementary materials are available at *Open Forum Infectious Diseases* online. Consisting of data provided by the authors to benefit the reader, the posted materials are not copyedited and are the sole responsibility of the authors, so questions or comments should be addressed to the corresponding author.
