# Supplementary material for: Estimated Deaths Averted in Adults by COVID-19 Vaccination in Select Latin American and Caribbean Countries
Source: Open Forum Infect Dis. 2024 Sep 10;11(10):ofae528. doi: 10.1093/ofid/ofae528 (PMC11474601; doi:10.1093/ofid/ofae528)
Supplement: ofae528_Supplementary_Data [file ofae528_supplementary_data.pdf]

Supplementary Figure 1.

Observed deaths (solid line) and model estimates for deaths without vaccination (dashed line) by age-group (18-59: left , 60+: right panel) over time. A. Cumulative deaths over time, B. Incident deaths over time. No correction for underreporting of COVID-19 deaths.

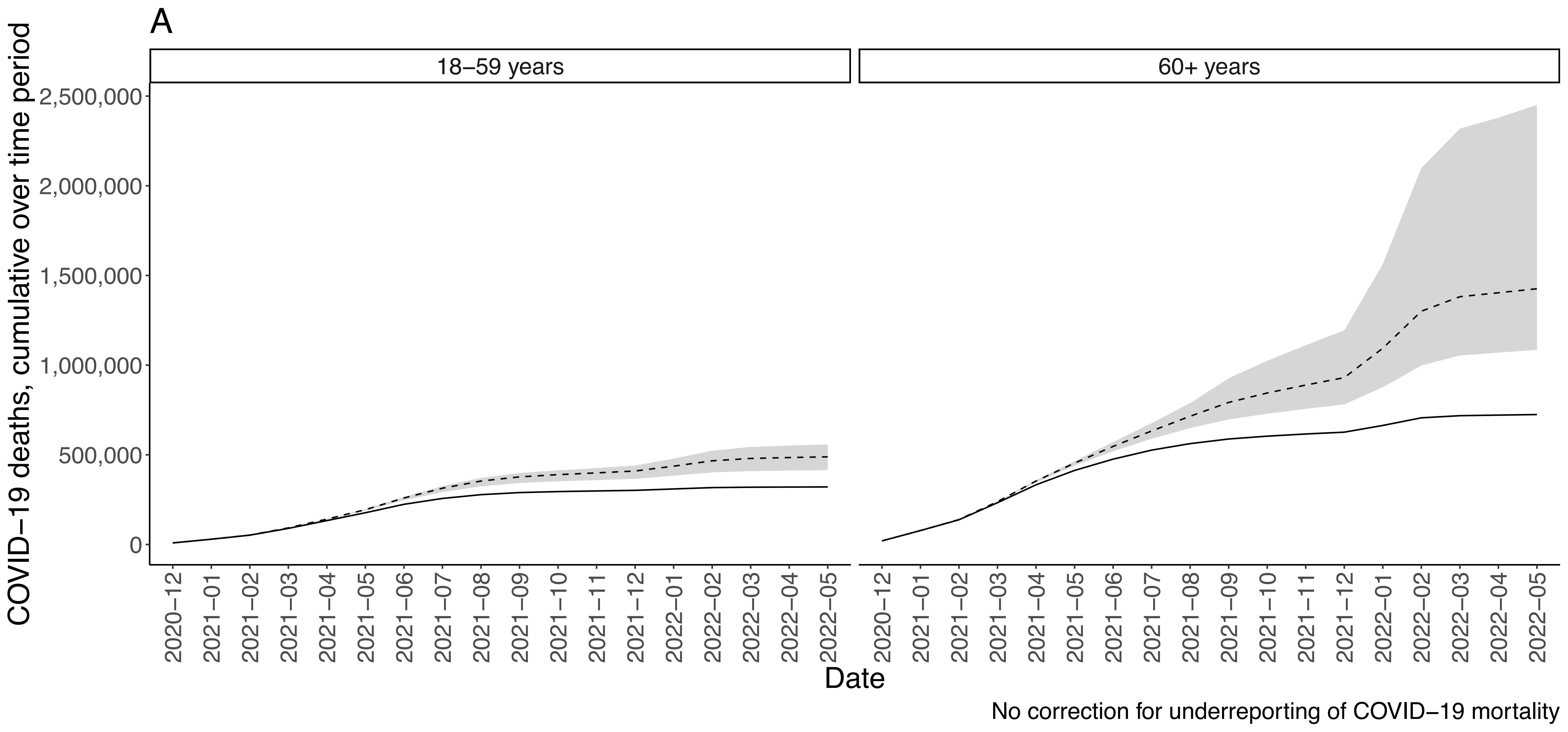
A
18–59 years
60+ years
COVID−19 deaths, cumulative over time period
2,500,000
2,000,000
1,500,000
1,000,000
500,000
0
2020−12
2021−01
2021−02
2021−03
2021−04
2021−05
2021−06
2021−07
2021−08
2021−09
2021−10
2021−11
2021−12
2022−01
2022−02
2022−03
2022−04
2022−05
Date
No correction for underreporting of COVID−19 mortality

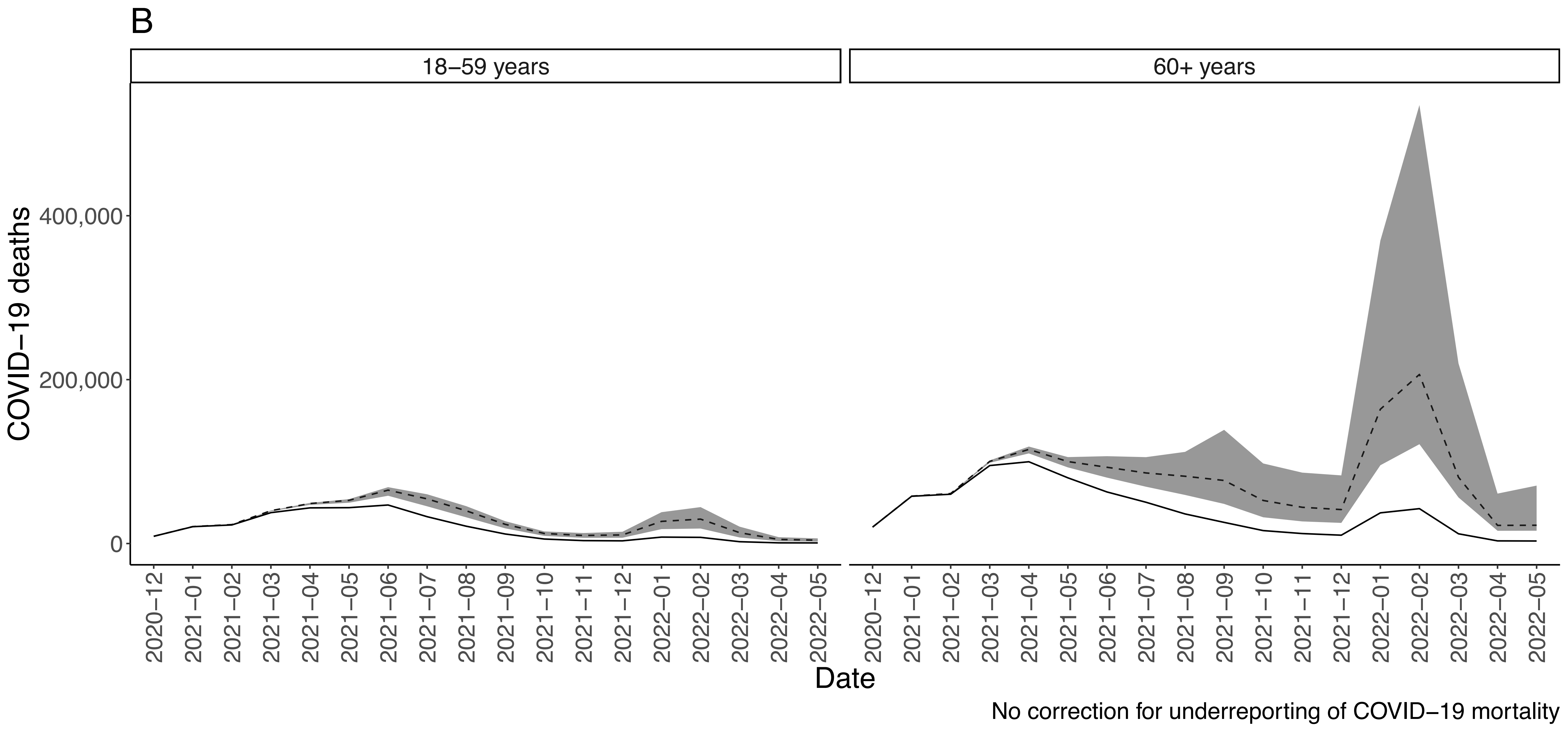

B
18–59 years
60+ years
COVID–19 deaths
400,000
200,000
0
2020–12
2021–01
2021–02
2021–03
2021–04
2021–05
2021–06
2021–07
2021–08
2021–09
2021–10
2021–11
2021–12
2022–01
2022–02
2022–03
2022–04
2022–05
Date
No correction for underreporting of COVID–19 mortality

Supplementary Figure 2.

Incident observed deaths, per 100,000 population, (solid line) and model estimates for deaths without vaccination, per 100,000, (dashed line) by age-group (18-59: left , 60+: right panel) over time, by country.

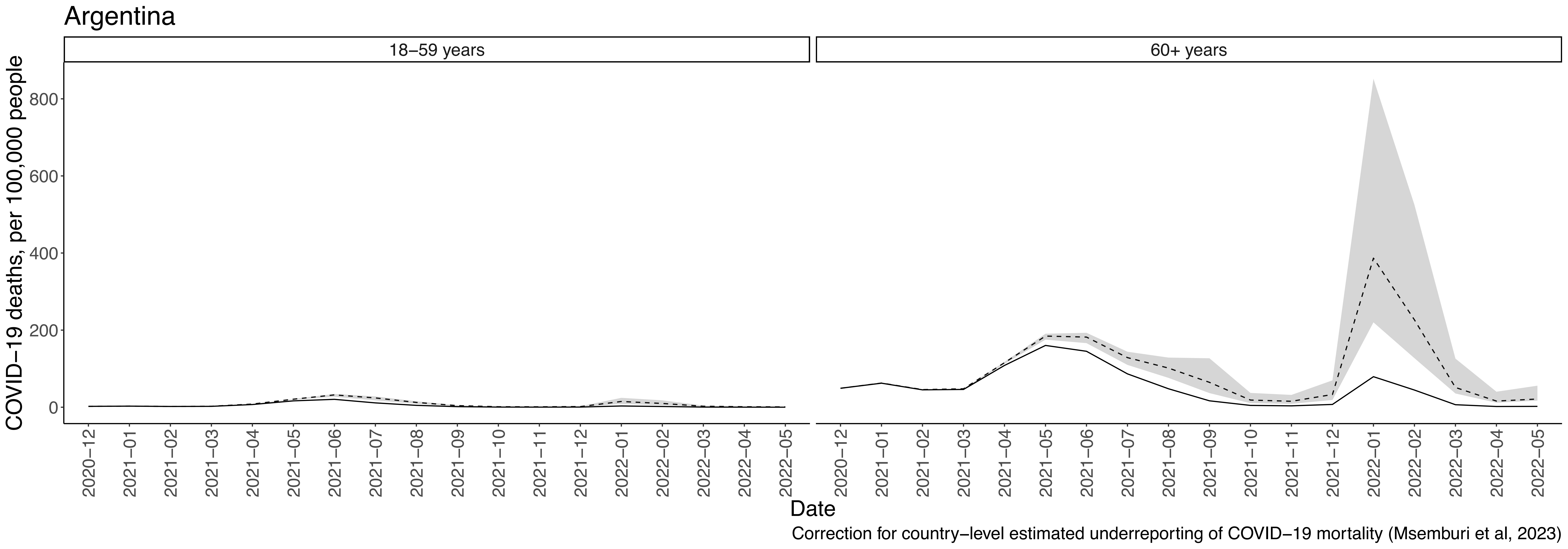

Argentina
18–59 years
60+ years
COVID−19 deaths, per 100,000 people
0
200
400
600
800
2020−12
2021−01
2021−02
2021−03
2021−04
2021−05
2021−06
2021−07
2021−08
2021−09
2021−10
2021−11
2021−12
2022−01
2022−02
2022−03
2022−04
2022−05
Date
Correction for country−level estimated underreporting of COVID−19 mortality (Msemburi et al, 2023)

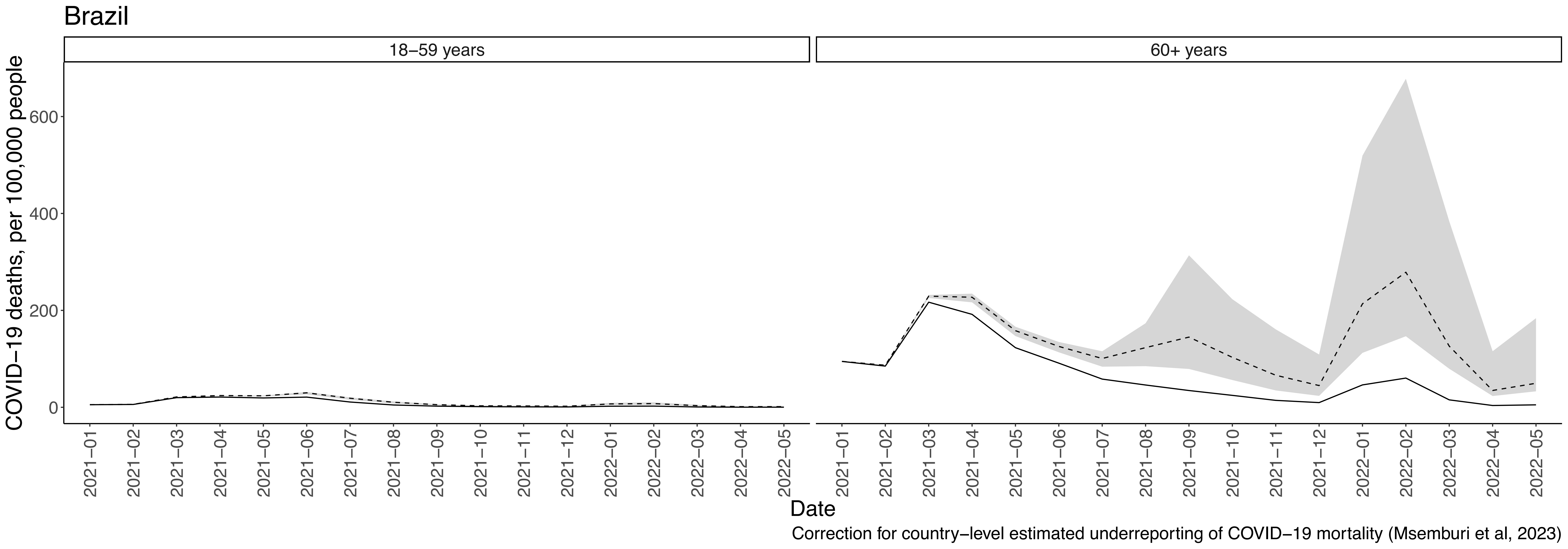

Brazil
18–59 years
60+ years
COVID−19 deaths, per 100,000 people
0
200
400
600
2021−01
2021−02
2021−03
2021−04
2021−05
2021−06
2021−07
2021−08
2021−09
2021−10
2021−11
2021−12
2022−01
2022−02
2022−03
2022−04
2022−05
Date
Correction for country−level estimated underreporting of COVID−19 mortality (Msemburi et al, 2023)

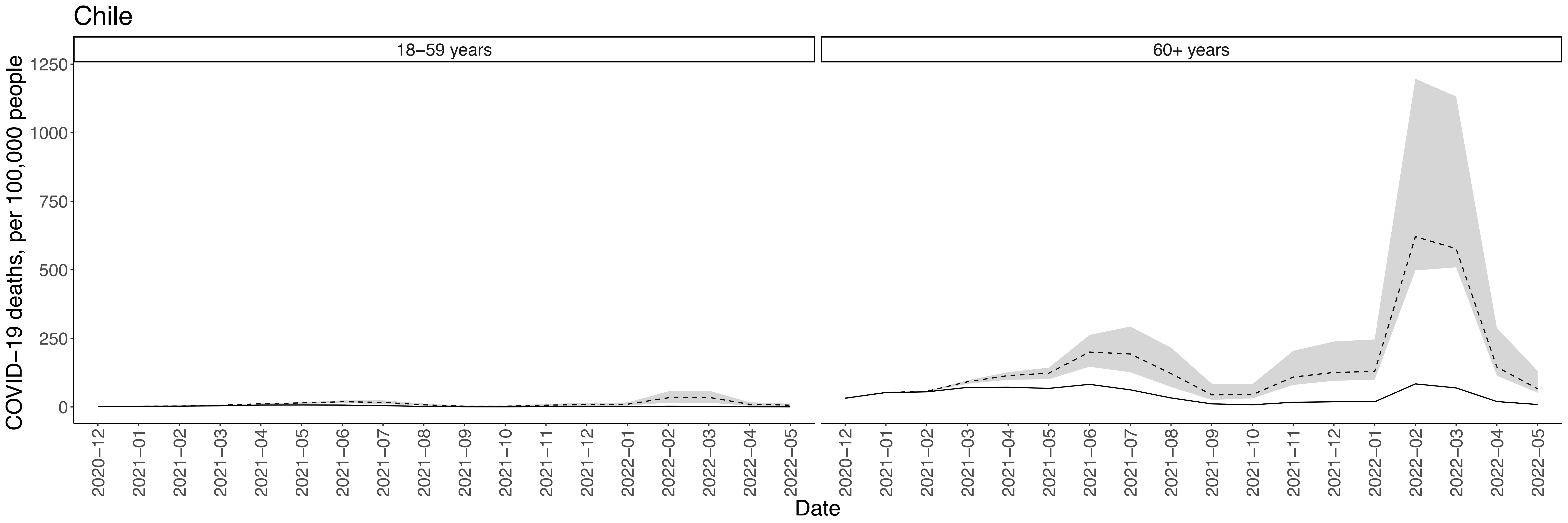

Correction for country−level estimated underreporting of COVID−19 mortality (Msemburi et al, 2023)

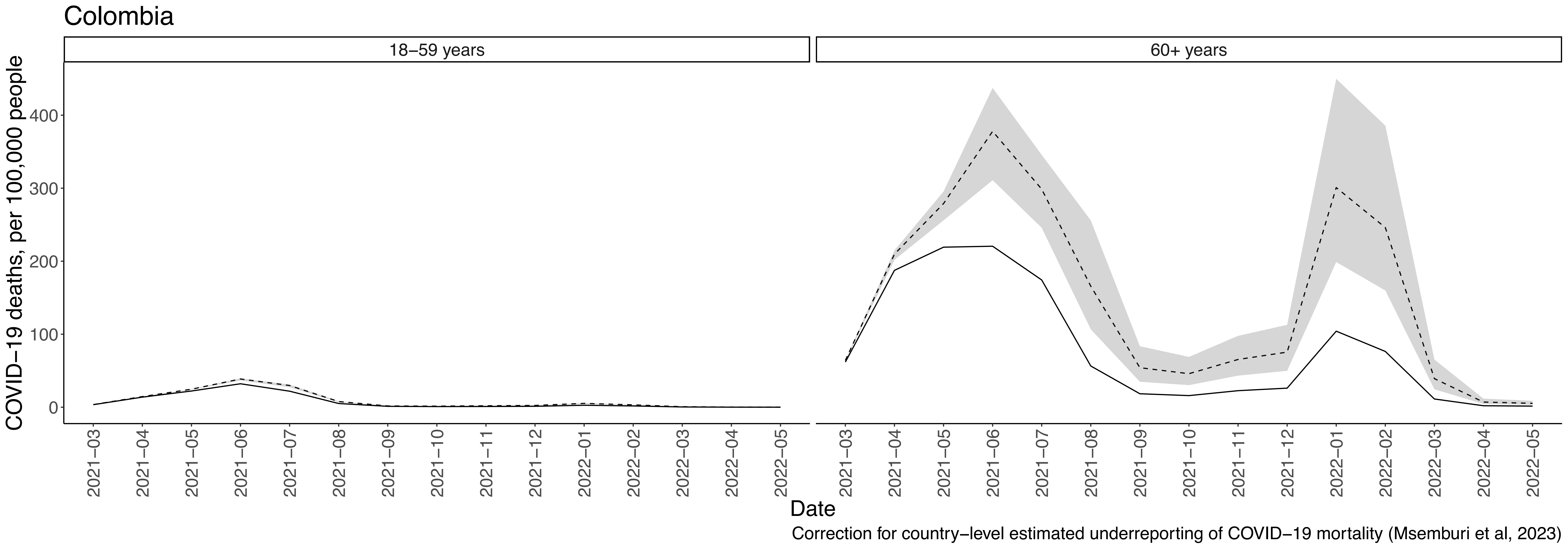

Colombia
18–59 years
60+ years
COVID−19 deaths, per 100,000 people
0
100
200
300
400
2021−03
2021−04
2021−05
2021−06
2021−07
2021−08
2021−09
2021−10
2021−11
2021−12
2022−01
2022−02
2022−03
2022−04
2022−05
Date
Correction for country−level estimated underreporting of COVID−19 mortality (Msemburi et al, 2023)

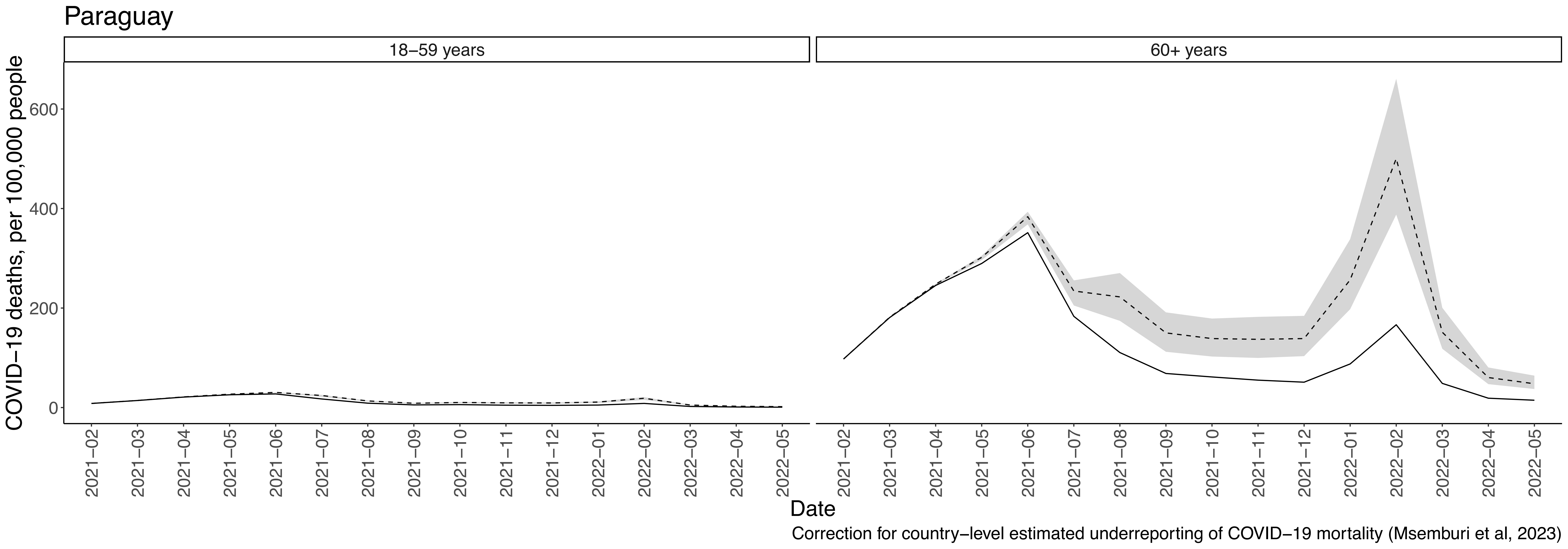
Paraguay
18–59 years
60+ years
COVID−19 deaths, per 100,000 people
0
200
400
600
2021−02
2021−03
2021−04
2021−05
2021−06
2021−07
2021−08
2021−09
2021−10
2021−11
2021−12
2022−01
2022−02
2022−03
2022−04
2022−05
Date
Correction for country−level estimated underreporting of COVID−19 mortality (Msemburi et al, 2023)

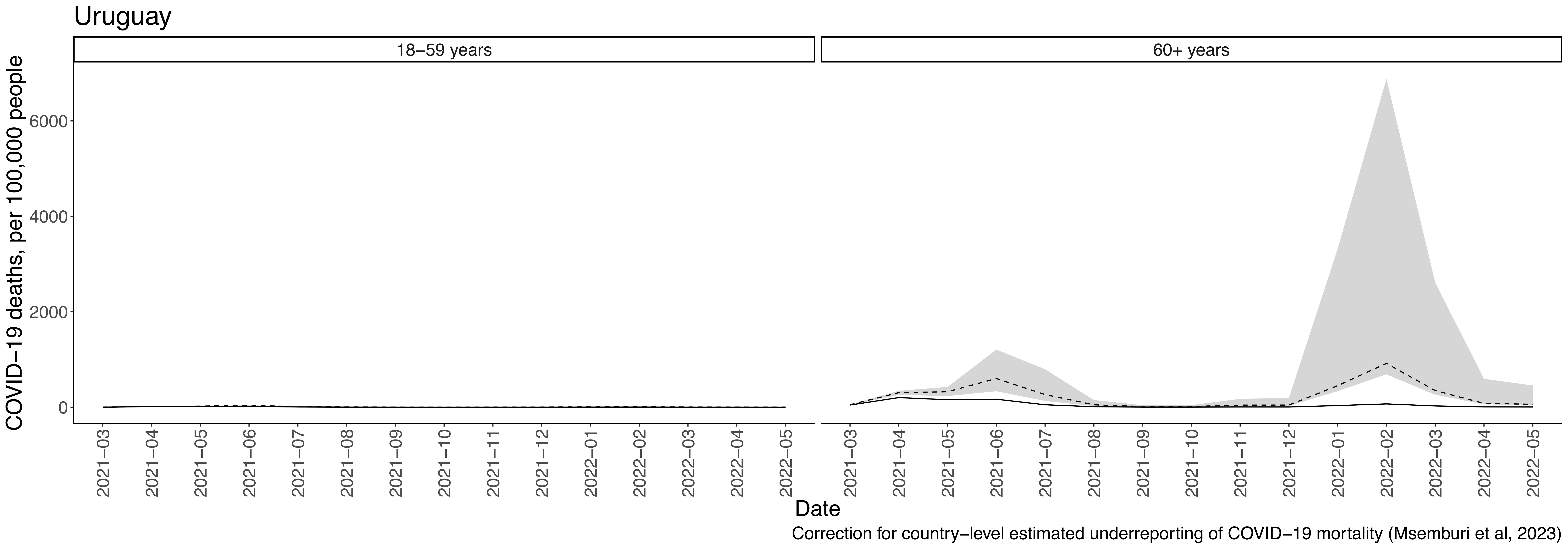
Uruguay
18–59 years
60+ years
COVID−19 deaths, per 100,000 people
6000
4000
2000
0
2021−03
2021−04
2021−05
2021−06
2021−07
2021−08
2021−09
2021−10
2021−11
2021−12
2022−01
2022−02
2022−03
2022−04
2022−05
Date
Correction for country−level estimated underreporting of COVID−19 mortality (Msemburi et al, 2023)

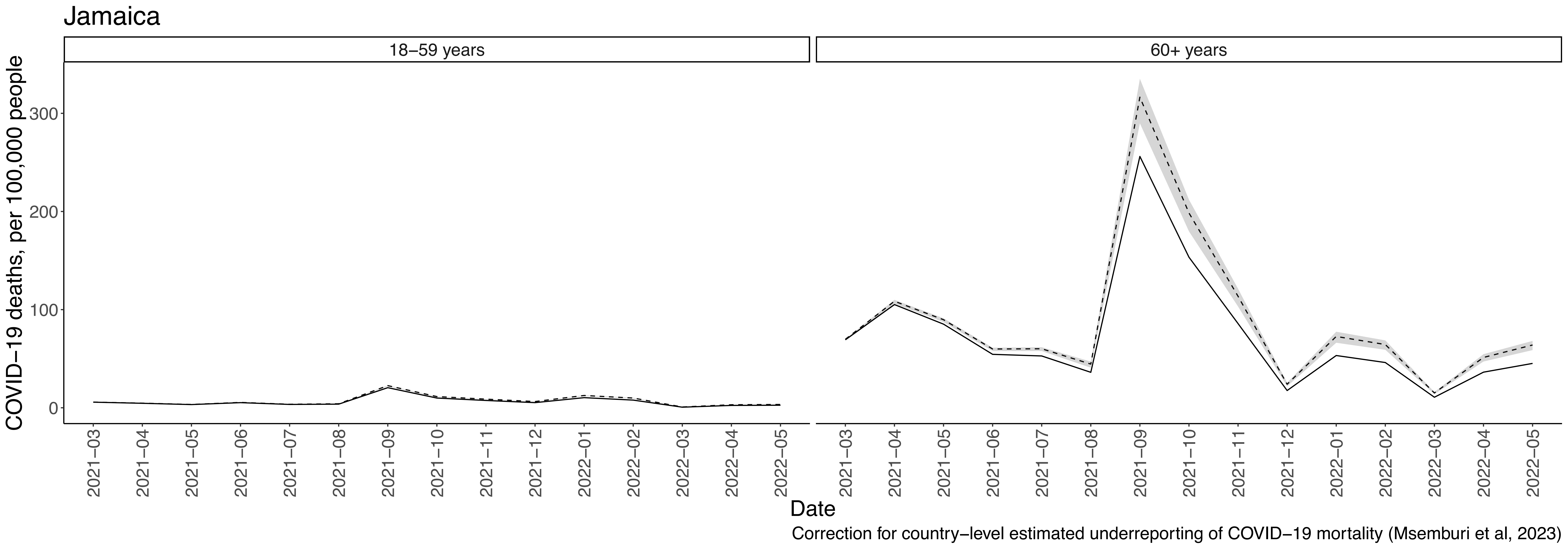
Jamaica
18–59 years
60+ years
COVID−19 deaths, per 100,000 people
0
100
200
300
2021−03
2021−04
2021−05
2021−06
2021−07
2021−08
2021−09
2021−10
2021−11
2021−12
2022−01
2022−02
2022−03
2022−04
2022−05
Date
Correction for country−level estimated underreporting of COVID−19 mortality (Msemburi et al, 2023)

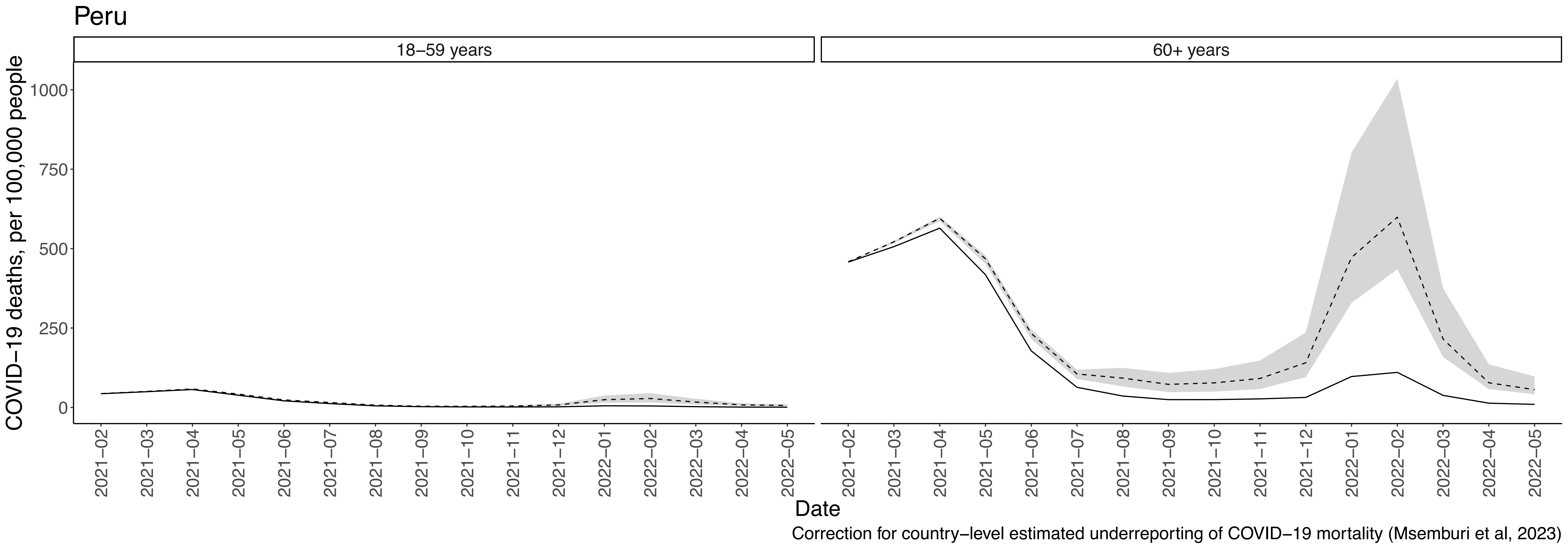
Peru
18–59 years
60+ years
COVID−19 deaths, per 100,000 people
1000
750
500
250
0
2021−02
2021−03
2021−04
2021−05
2021−06
2021−07
2021−08
2021−09
2021−10
2021−11
2021−12
2022−01
2022−02
2022−03
2022−04
2022−05
Date
Correction for country−level estimated underreporting of COVID−19 mortality (Msemburi et al, 2023)

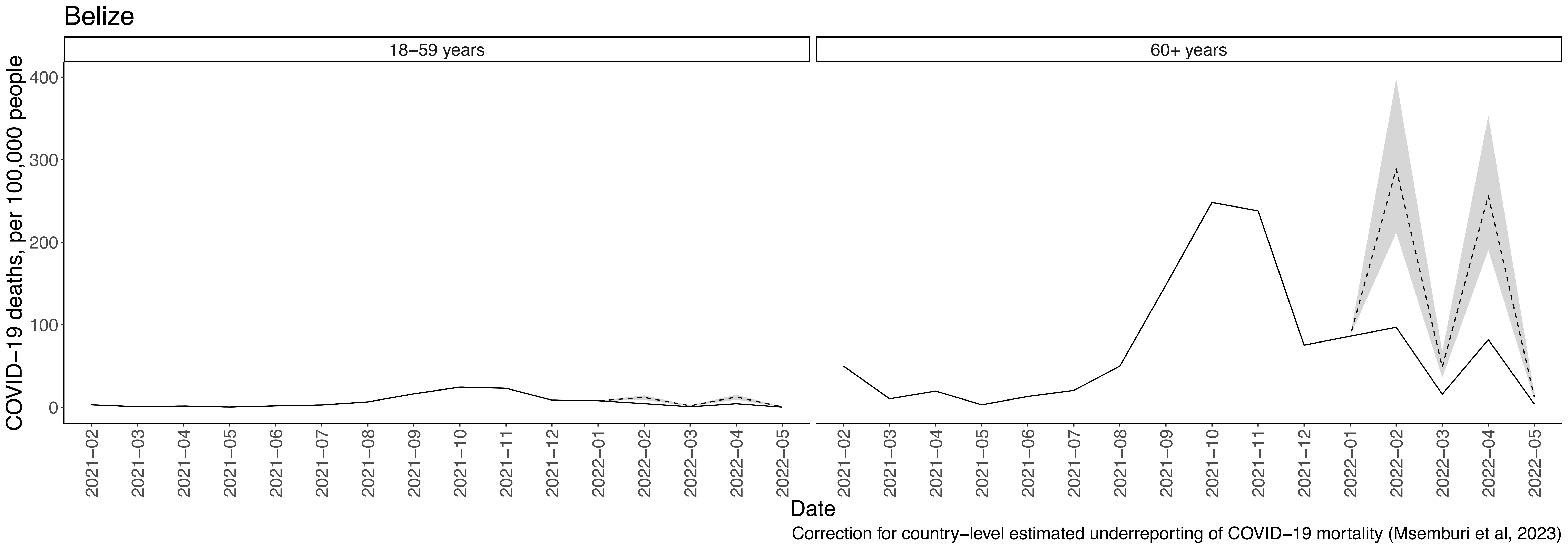

Belize
18–59 years
60+ years
COVID−19 deaths, per 100,000 people
400
300
200
100
0
2021−02
2021−03
2021−04
2021−05
2021−06
2021−07
2021−08
2021−09
2021−10
2021−11
2021−12
2022−01
2022−02
2022−03
2022−04
2022−05
Date
Correction for country−level estimated underreporting of COVID−19 mortality (Msemburi et al, 2023)

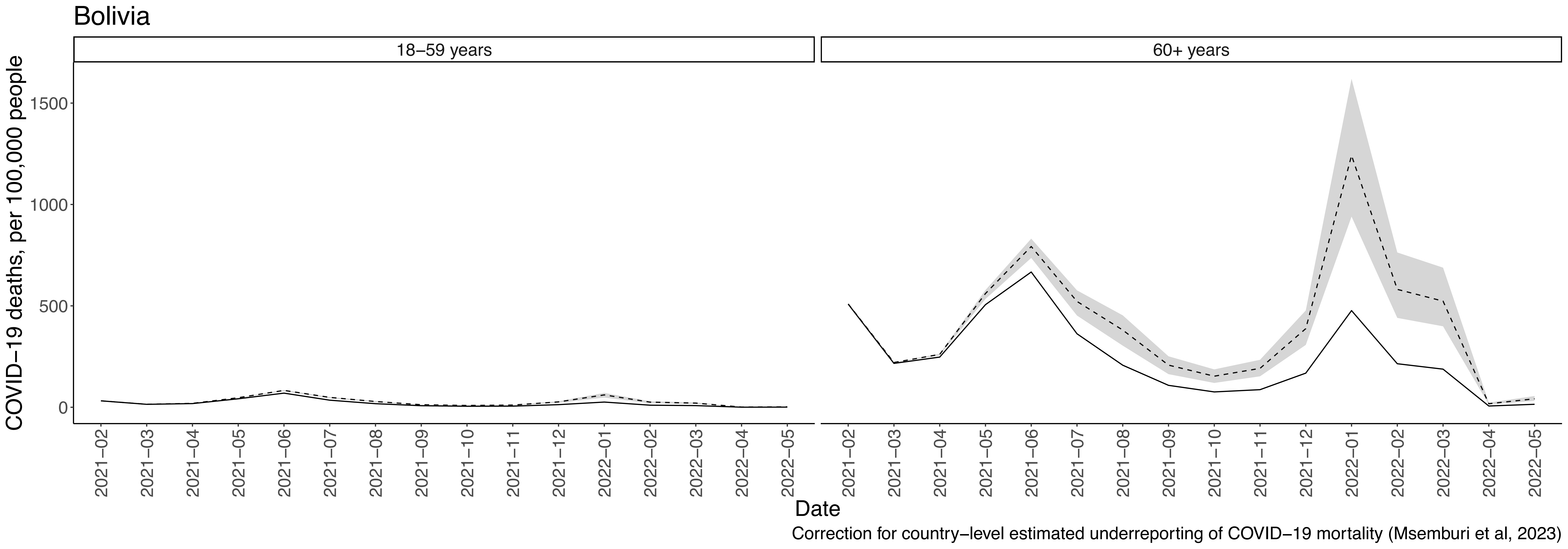
Bolivia
18–59 years
60+ years
COVID−19 deaths, per 100,000 people
0
500
1000
1500
2021−02
2021−03
2021−04
2021−05
2021−06
2021−07
2021−08
2021−09
2021−10
2021−11
2021−12
2022−01
2022−02
2022−03
2022−04
2022−05
Date
Correction for country−level estimated underreporting of COVID−19 mortality (Msemburi et al, 2023)

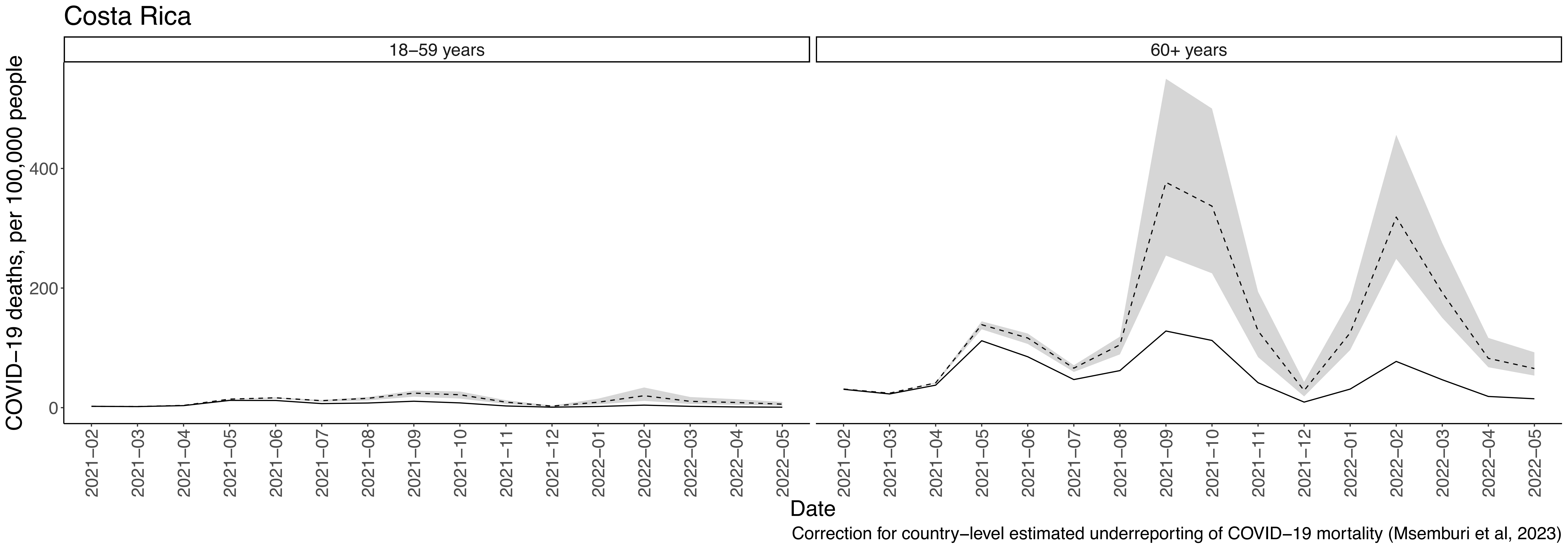

Costa Rica
18–59 years
60+ years
COVID–19 deaths, per 100,000 people
0
200
400
2021–02
2021–03
2021–04
2021–05
2021–06
2021–07
2021–08
2021–09
2021–10
2021–11
2021–12
2022–01
2022–02
2022–03
2022–04
2022–05
Date
Correction for country–level estimated underreporting of COVID–19 mortality (Msemburi et al, 2023)

Ecuador

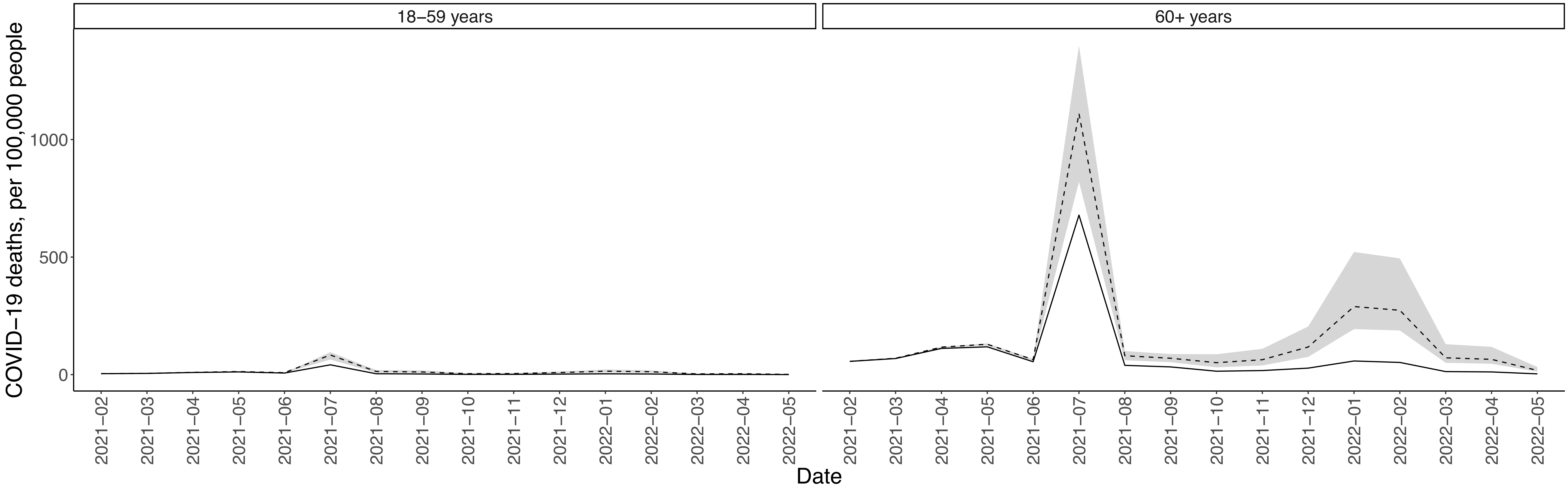

Correction for country−level estimated underreporting of COVID−19 mortality (Msemburi et al, 2023)

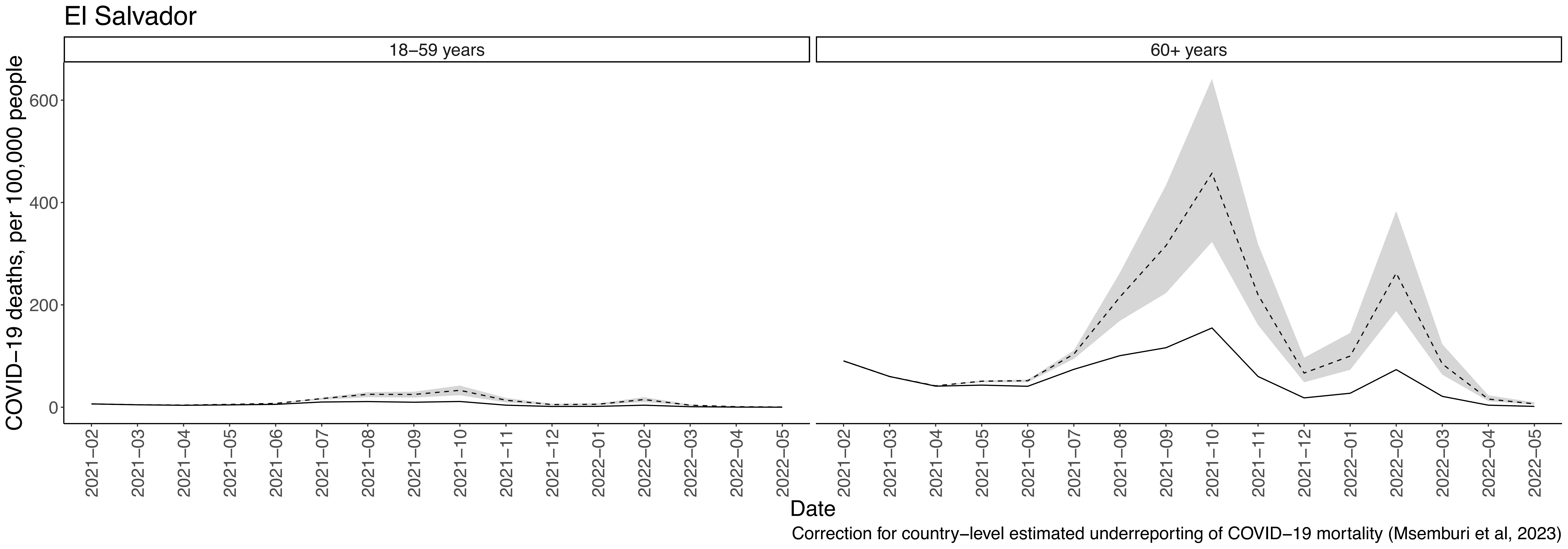

El Salvador
18–59 years
60+ years
COVID−19 deaths, per 100,000 people
0
200
400
600
2021−02
2021−03
2021−04
2021−05
2021−06
2021−07
2021−08
2021−09
2021−10
2021−11
2021−12
2022−01
2022−02
2022−03
2022−04
2022−05
Date
Correction for country−level estimated underreporting of COVID−19 mortality (Msemburi et al, 2023)

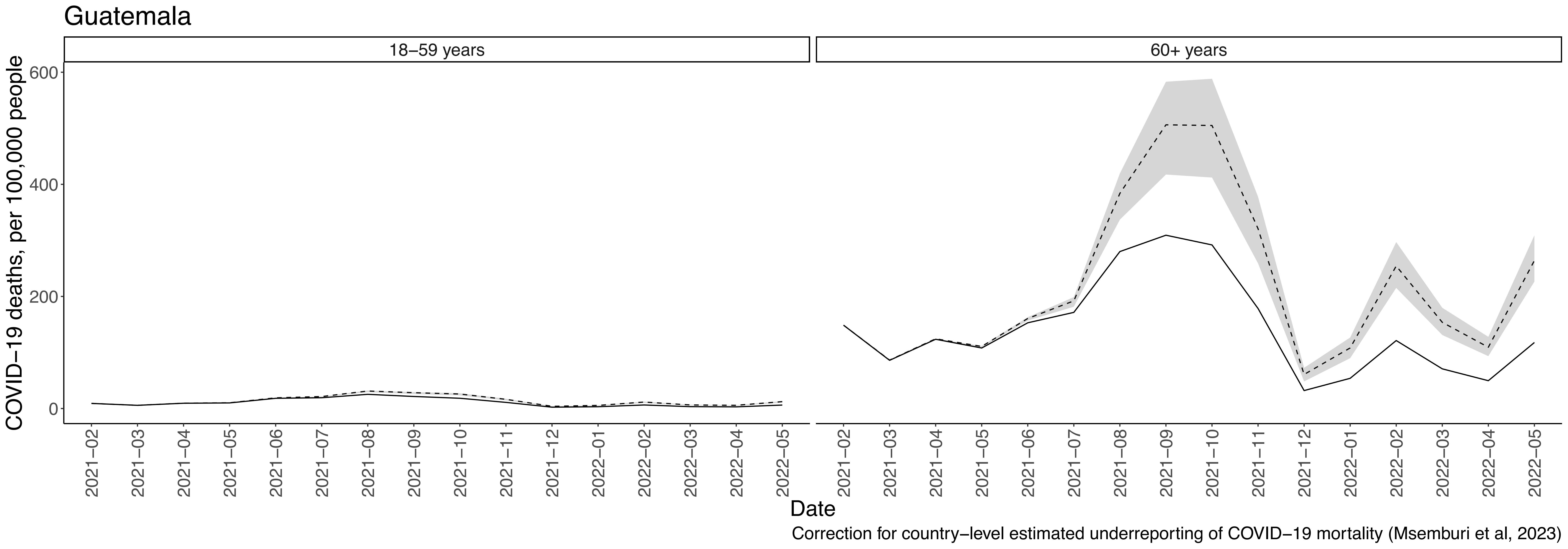
Guatemala
18–59 years
60+ years
COVID−19 deaths, per 100,000 people
0
200
400
600
2021−02
2021−03
2021−04
2021−05
2021−06
2021−07
2021−08
2021−09
2021−10
2021−11
2021−12
2022−01
2022−02
2022−03
2022−04
2022−05
Date
Correction for country−level estimated underreporting of COVID−19 mortality (Msemburi et al, 2023)

Honduras

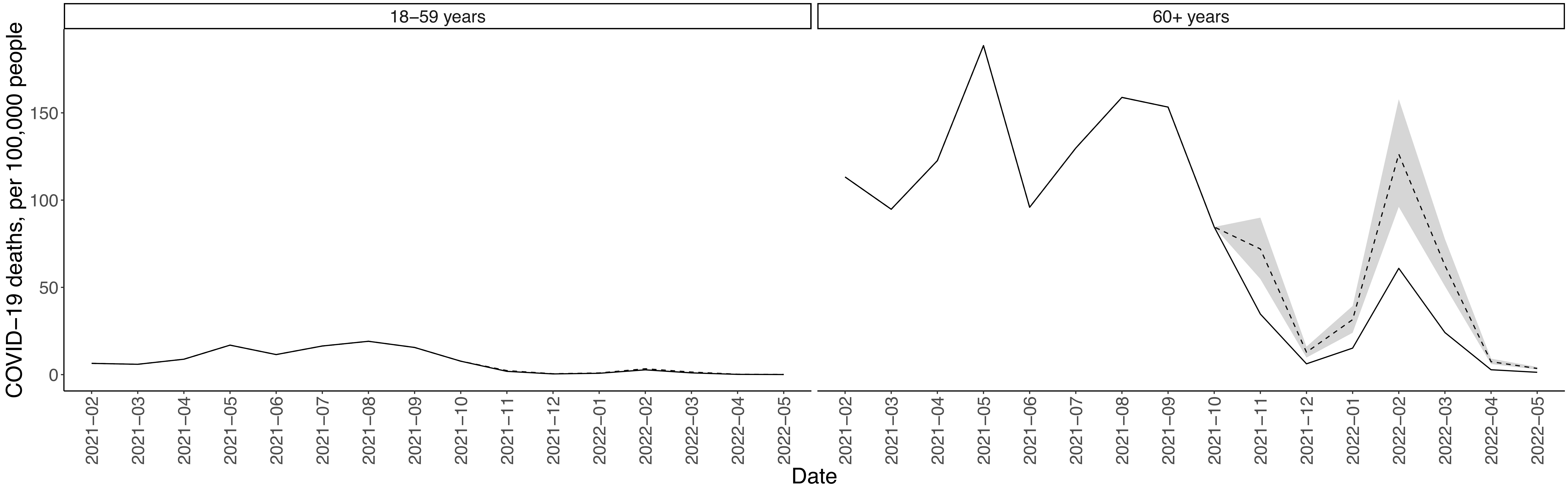

Correction for country−level estimated underreporting of COVID−19 mortality (Msemburi et al, 2023)

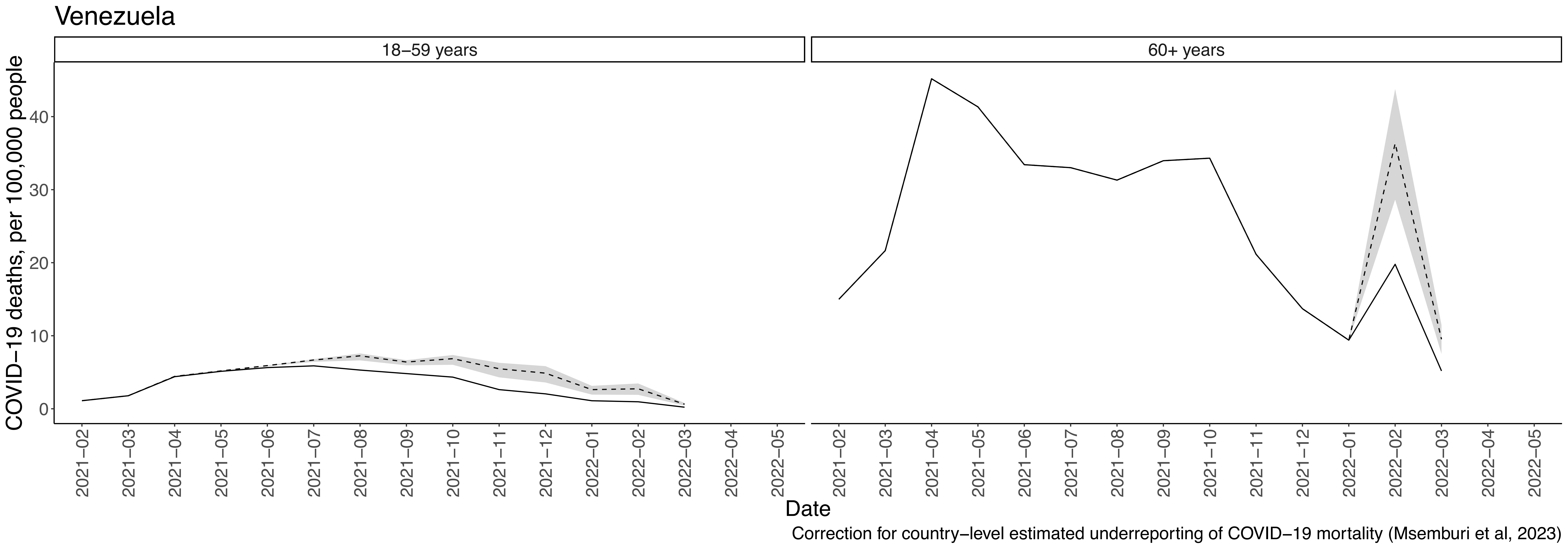

Venezuela
18–59 years
60+ years
COVID−19 deaths, per 100,000 people
0
10
20
30
40
2021−02
2021−03
2021−04
2021−05
2021−06
2021−07
2021−08
2021−09
2021−10
2021−11
2021−12
2022−01
2022−02
2022−03
2022−04
2022−05
Date
Correction for country−level estimated underreporting of COVID−19 mortality (Msemburi et al, 2023)

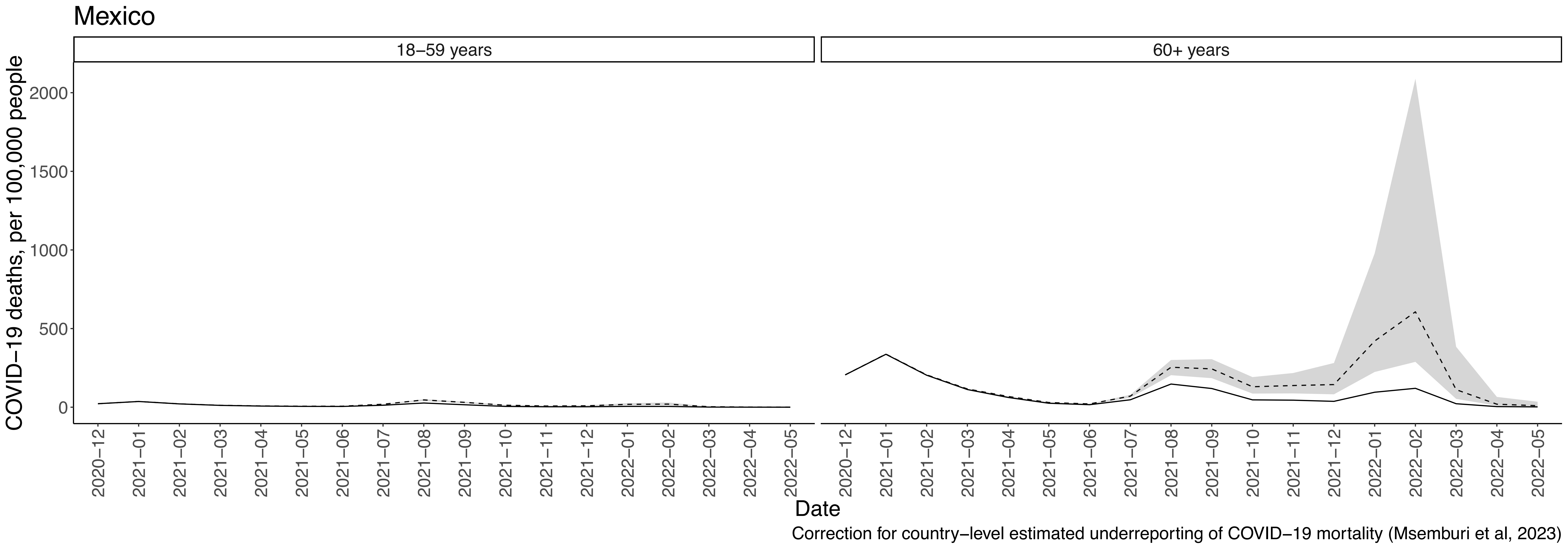

Mexico
18–59 years
60+ years
COVID−19 deaths, per 100,000 people
0
500
1000
1500
2000
2020−12
2021−01
2021−02
2021−03
2021−04
2021−05
2021−06
2021−07
2021−08
2021−09
2021−10
2021−11
2021−12
2022−01
2022−02
2022−03
2022−04
2022−05
Date
Correction for country−level estimated underreporting of COVID−19 mortality (Msemburi et al, 2023)

Supplementary Figure 3.

Incident observed deaths, per 100,000 population, (solid line) and model estimates for deaths without vaccination, per 100,000, (dashed line) by age-group (18-59: left , 60+: right panel) over time, by country. No correction for underreporting of COVID-19 deaths.

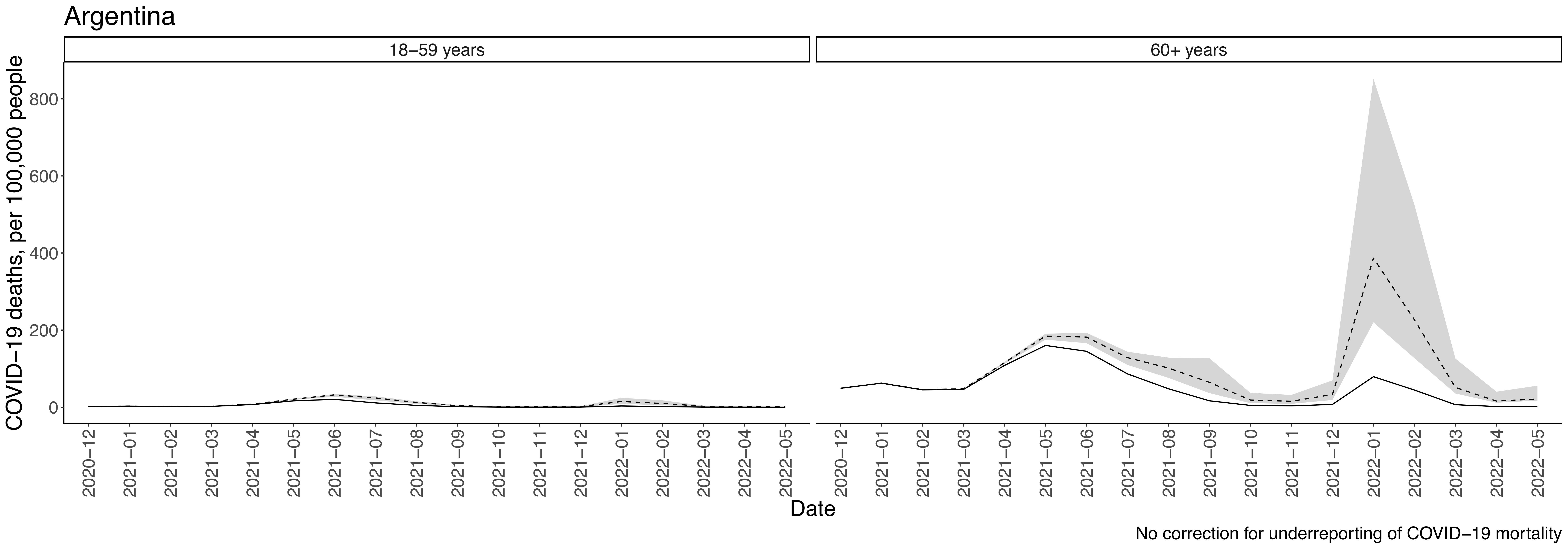
Argentina
18–59 years
60+ years
COVID−19 deaths, per 100,000 people
0
200
400
600
800
2020−12
2021−01
2021−02
2021−03
2021−04
2021−05
2021−06
2021−07
2021−08
2021−09
2021−10
2021−11
2021−12
2022−01
2022−02
2022−03
2022−04
2022−05
Date
No correction for underreporting of COVID−19 mortality

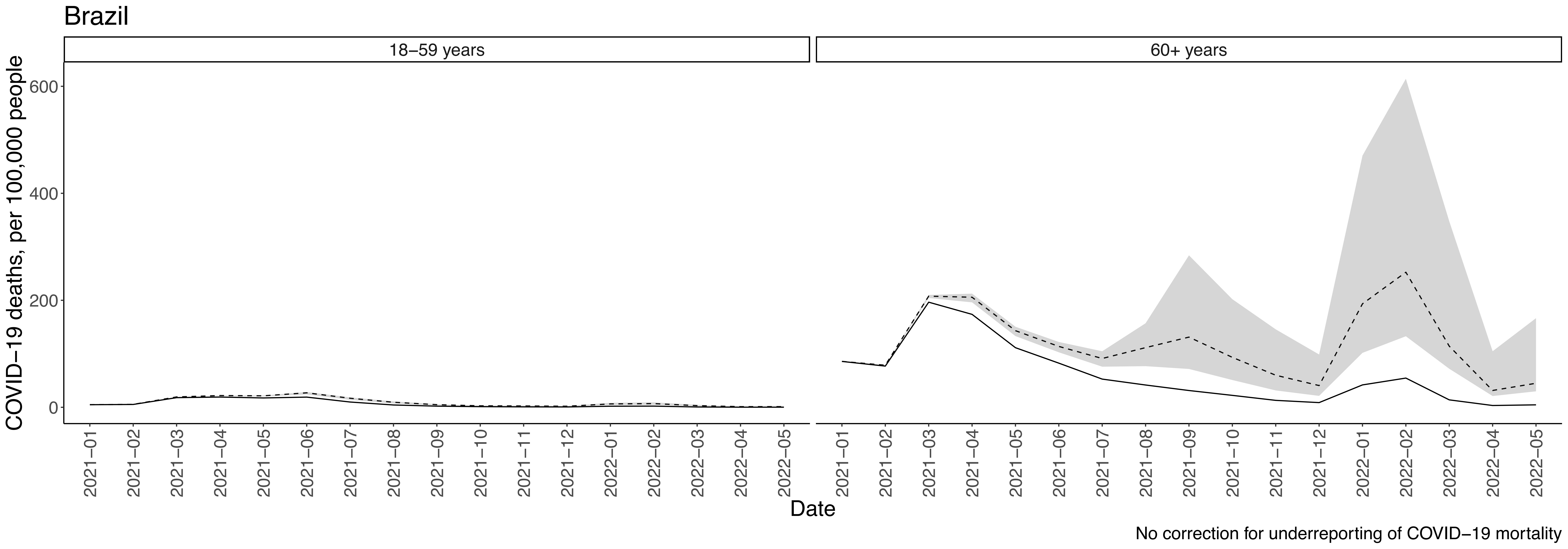

Brazil
18–59 years
60+ years
COVID−19 deaths, per 100,000 people
600
400
200
0
2021−01
2021−02
2021−03
2021−04
2021−05
2021−06
2021−07
2021−08
2021−09
2021−10
2021−11
2021−12
2022−01
2022−02
2022−03
2022−04
2022−05
Date
No correction for underreporting of COVID−19 mortality

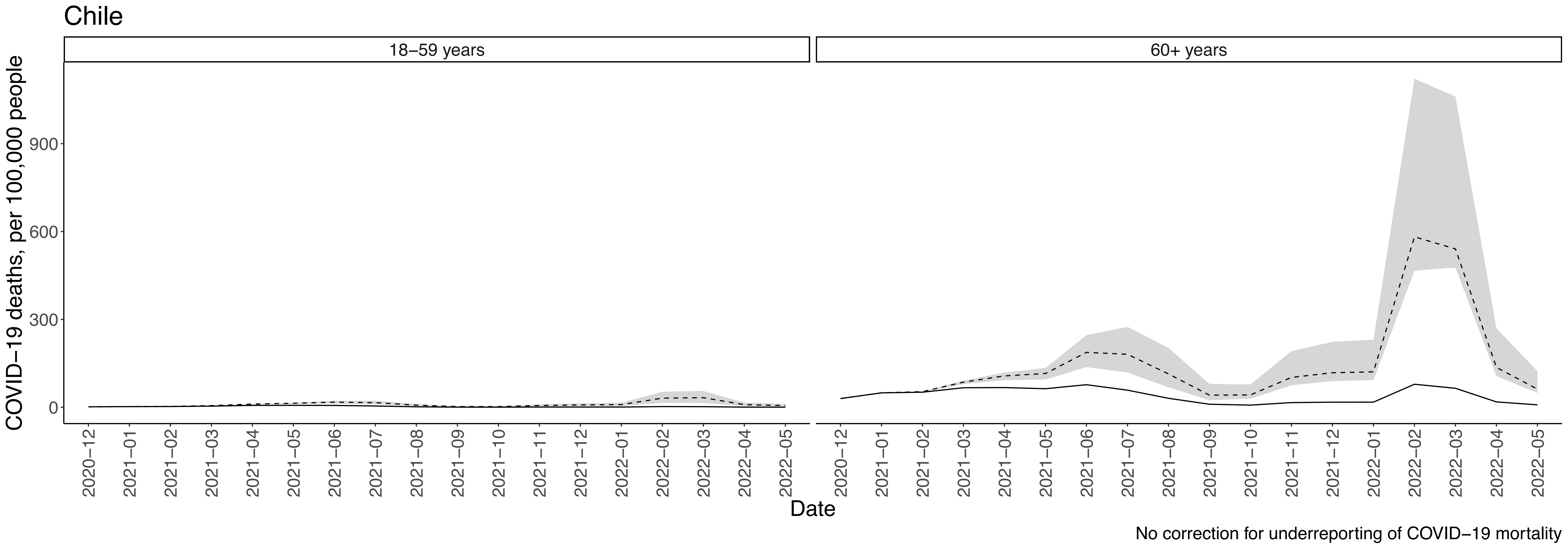

Chile
18–59 years
60+ years
COVID−19 deaths, per 100,000 people
900
600
300
0
2020−12
2021−01
2021−02
2021−03
2021−04
2021−05
2021−06
2021−07
2021−08
2021−09
2021−10
2021−11
2021−12
2022−01
2022−02
2022−03
2022−04
2022−05
Date
No correction for underreporting of COVID−19 mortality

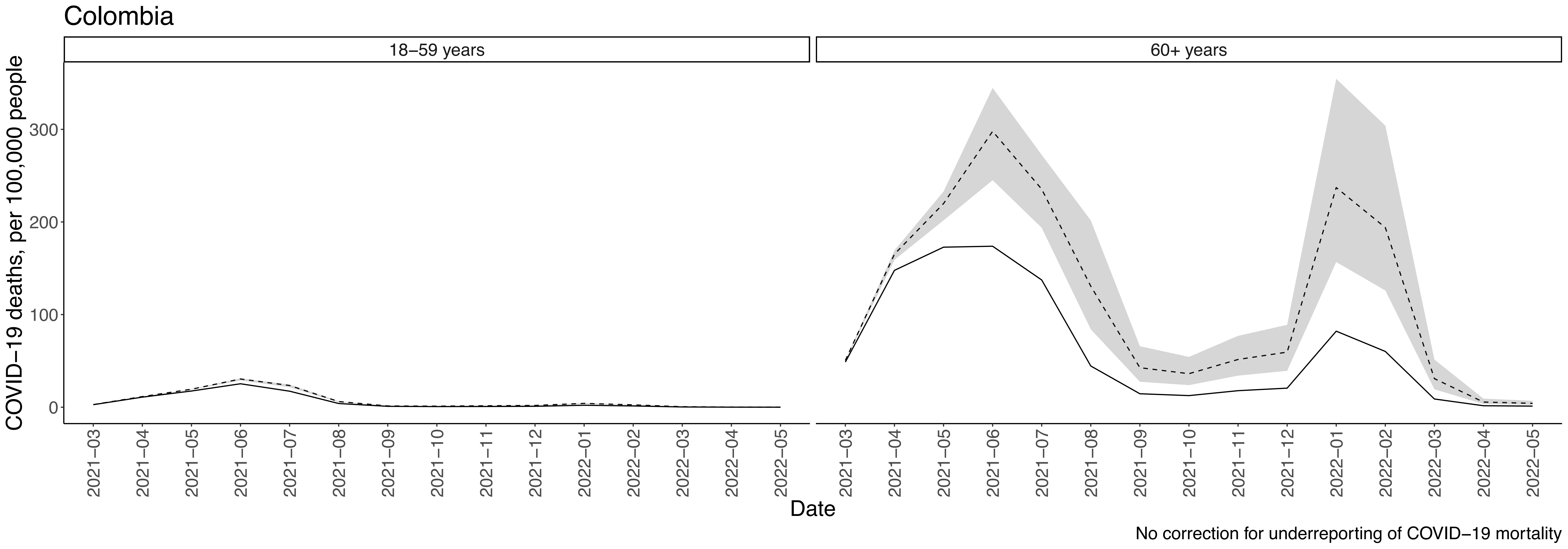

Colombia
18–59 years
60+ years
COVID−19 deaths, per 100,000 people
0
100
200
300
2021−03
2021−04
2021−05
2021−06
2021−07
2021−08
2021−09
2021−10
2021−11
2021−12
2022−01
2022−02
2022−03
2022−04
2022−05
Date
No correction for underreporting of COVID−19 mortality

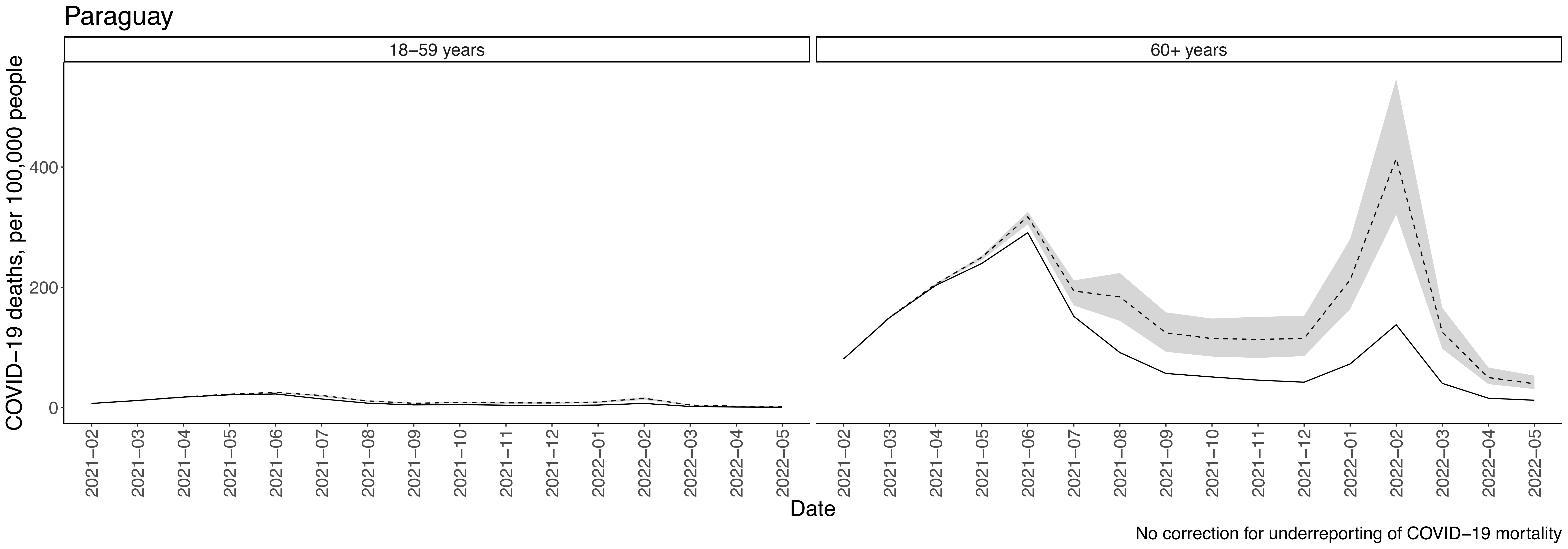

Paraguay
18–59 years
60+ years
COVID−19 deaths, per 100,000 people
0
200
400
2021−02
2021−03
2021−04
2021−05
2021−06
2021−07
2021−08
2021−09
2021−10
2021−11
2021−12
2022−01
2022−02
2022−03
2022−04
2022−05
Date
No correction for underreporting of COVID−19 mortality

Uruguay

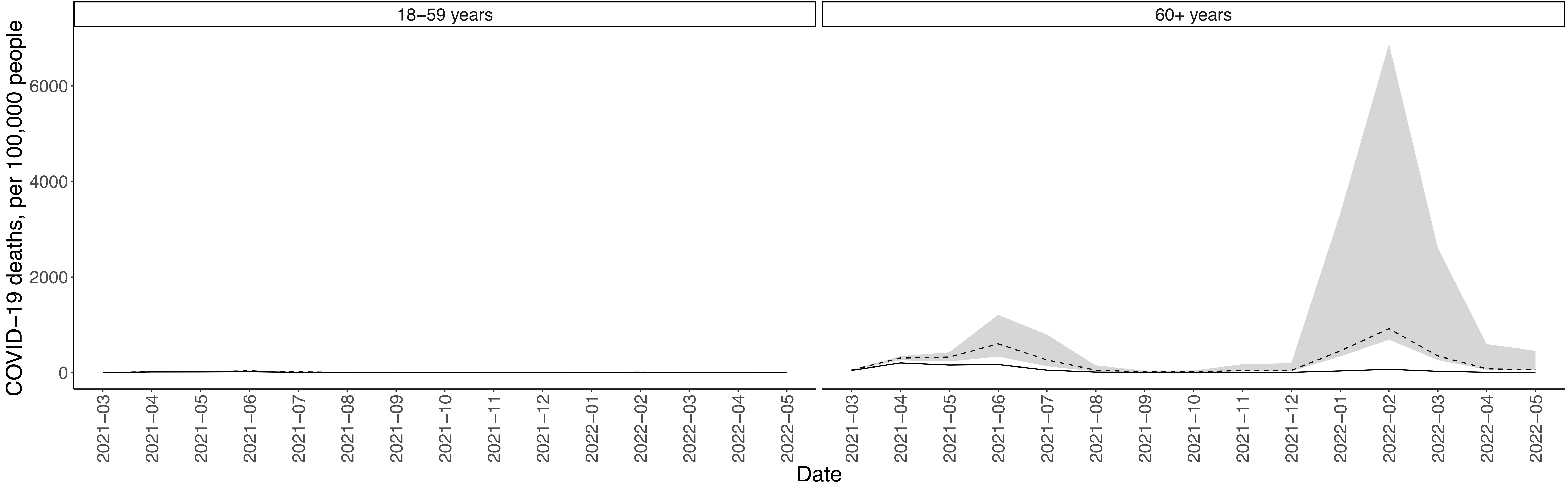

No correction for underreporting of COVID−19 mortality

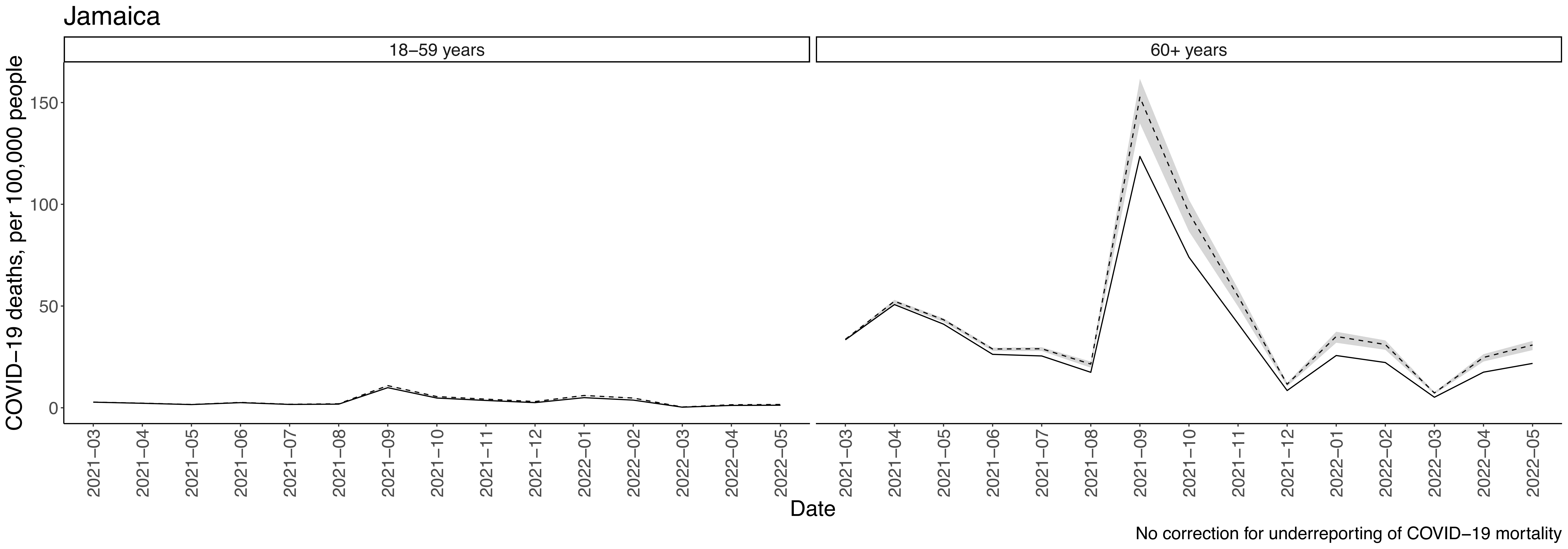
Jamaica
18–59 years
60+ years
COVID−19 deaths, per 100,000 people
0
50
100
150
2021−03
2021−04
2021−05
2021−06
2021−07
2021−08
2021−09
2021−10
2021−11
2021−12
2022−01
2022−02
2022−03
2022−04
2022−05
Date
No correction for underreporting of COVID−19 mortality

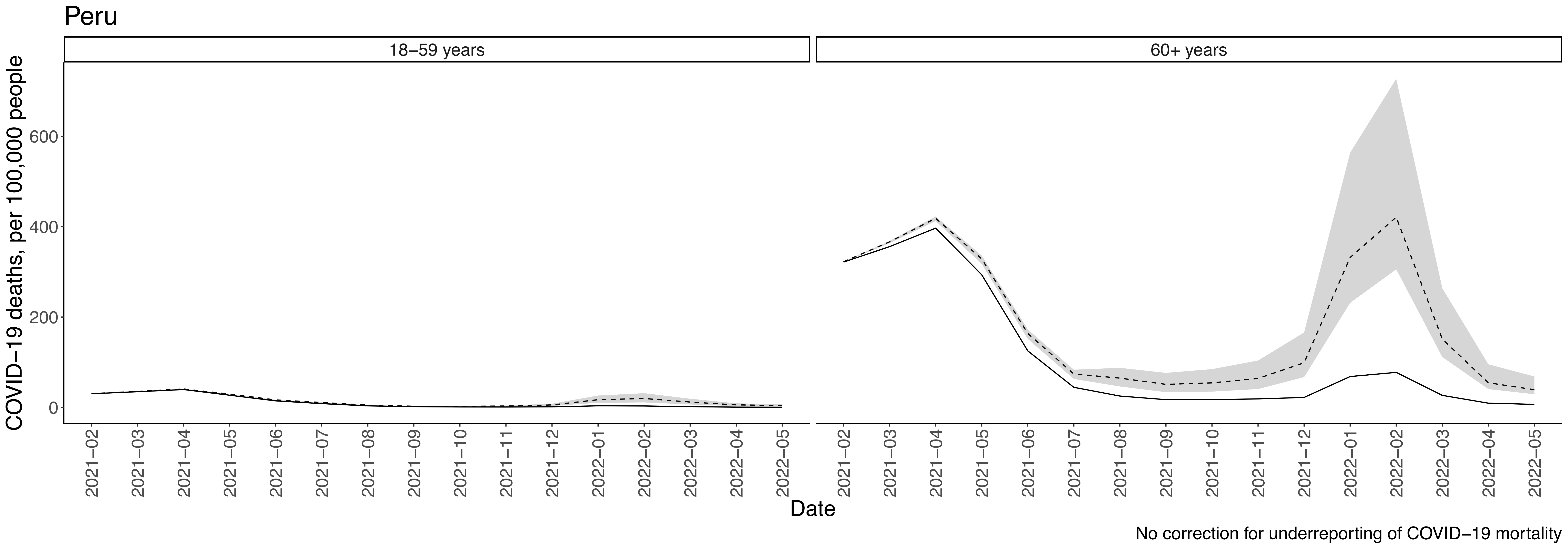

Peru
18–59 years
60+ years
COVID−19 deaths, per 100,000 people
0
200
400
600
2021−02
2021−03
2021−04
2021−05
2021−06
2021−07
2021−08
2021−09
2021−10
2021−11
2021−12
2022−01
2022−02
2022−03
2022−04
2022−05
Date
No correction for underreporting of COVID−19 mortality

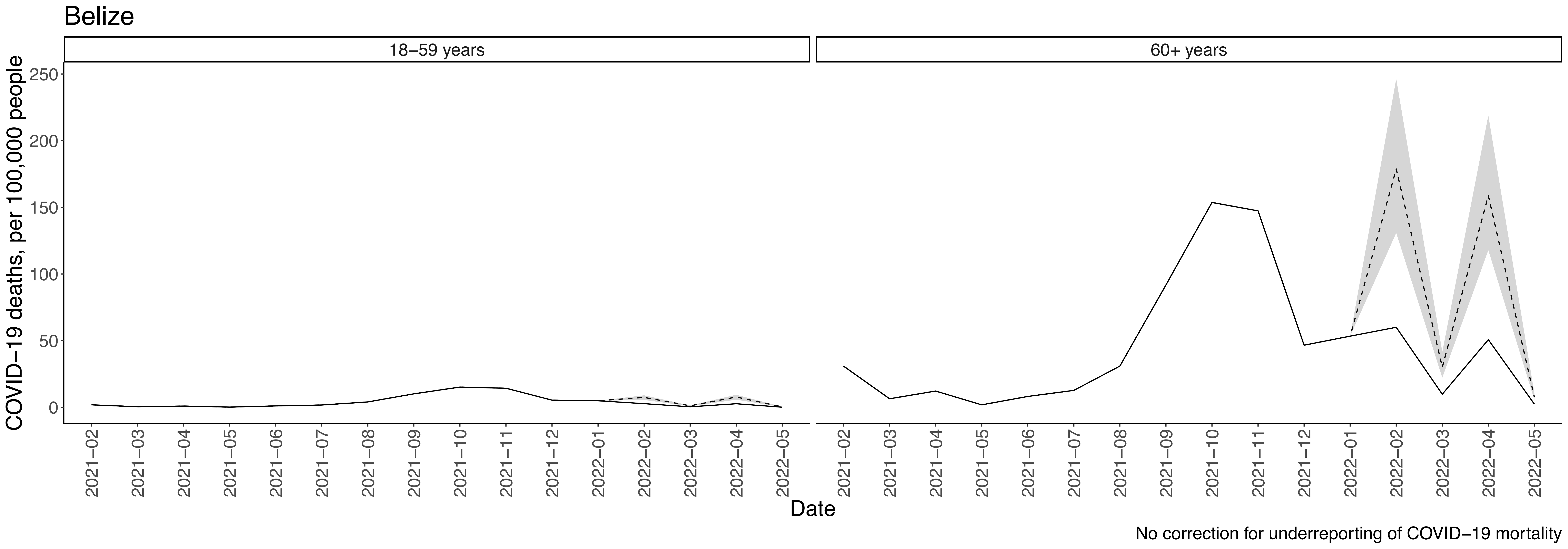

Belize
18–59 years
60+ years
COVID−19 deaths, per 100,000 people
0
50
100
150
200
250
2021−02
2021−03
2021−04
2021−05
2021−06
2021−07
2021−08
2021−09
2021−10
2021−11
2021−12
2022−01
2022−02
2022−03
2022−04
2022−05
Date
No correction for underreporting of COVID−19 mortality

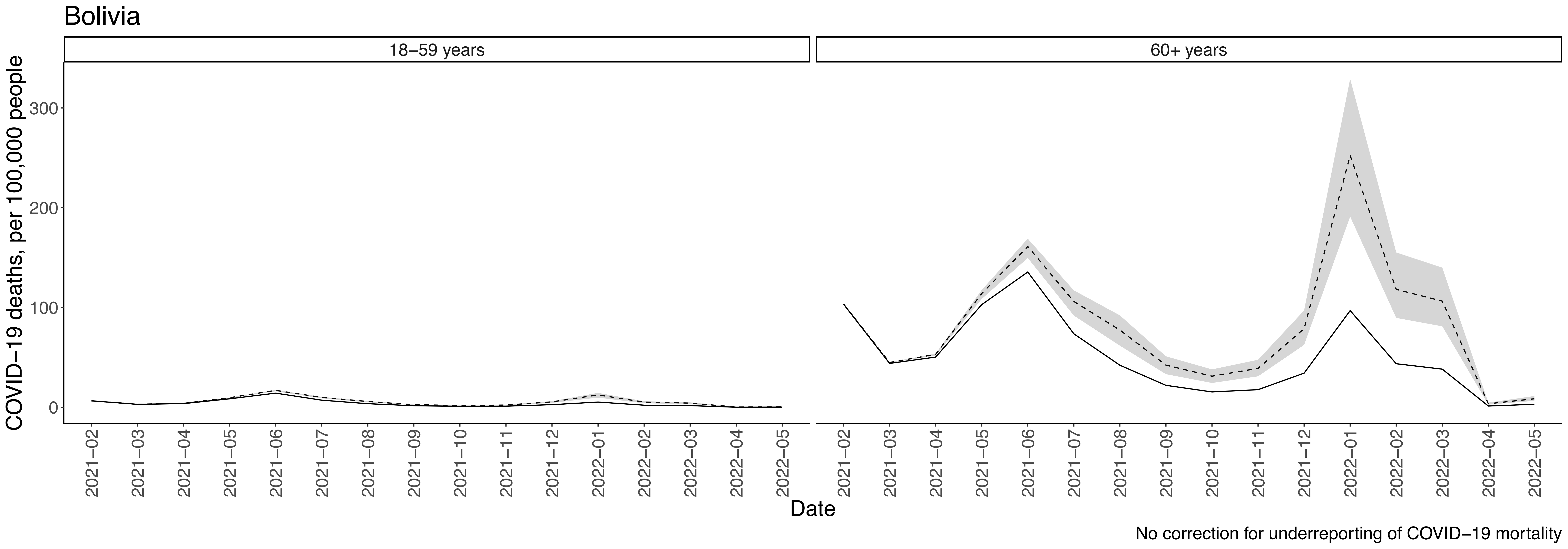
Bolivia
18–59 years
60+ years
COVID−19 deaths, per 100,000 people
0
100
200
300
2021−02
2021−03
2021−04
2021−05
2021−06
2021−07
2021−08
2021−09
2021−10
2021−11
2021−12
2022−01
2022−02
2022−03
2022−04
2022−05
Date
No correction for underreporting of COVID−19 mortality

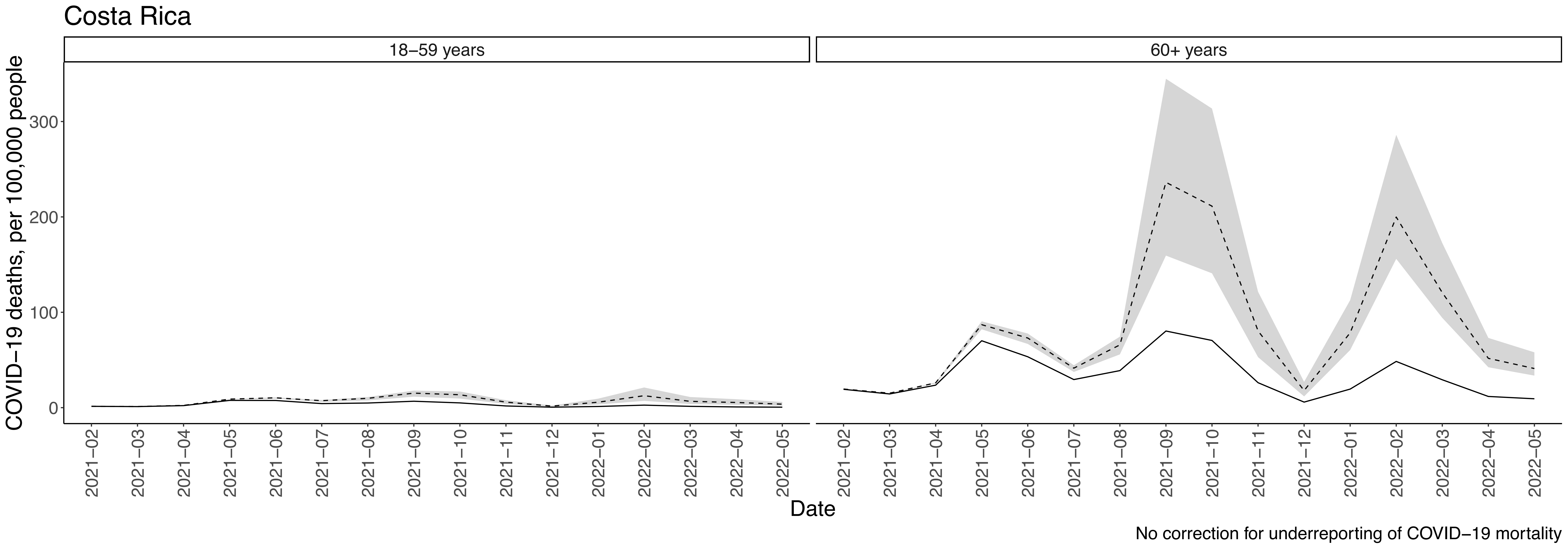

Costa Rica
18–59 years
60+ years
COVID−19 deaths, per 100,000 people
300
200
100
0
2021−02
2021−03
2021−04
2021−05
2021−06
2021−07
2021−08
2021−09
2021−10
2021−11
2021−12
2022−01
2022−02
2022−03
2022−04
2022−05
Date
No correction for underreporting of COVID−19 mortality

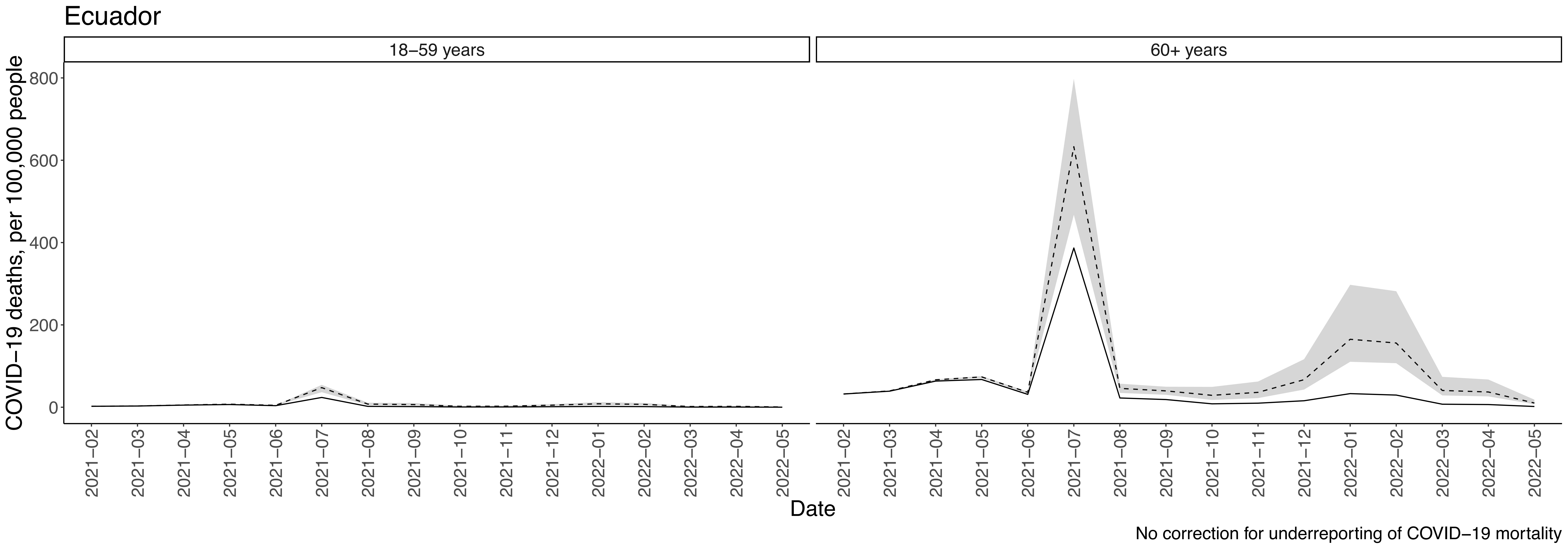

Ecuador
18–59 years
60+ years
COVID−19 deaths, per 100,000 people
0
200
400
600
800
2021−02
2021−03
2021−04
2021−05
2021−06
2021−07
2021−08
2021−09
2021−10
2021−11
2021−12
2022−01
2022−02
2022−03
2022−04
2022−05
Date
No correction for underreporting of COVID−19 mortality

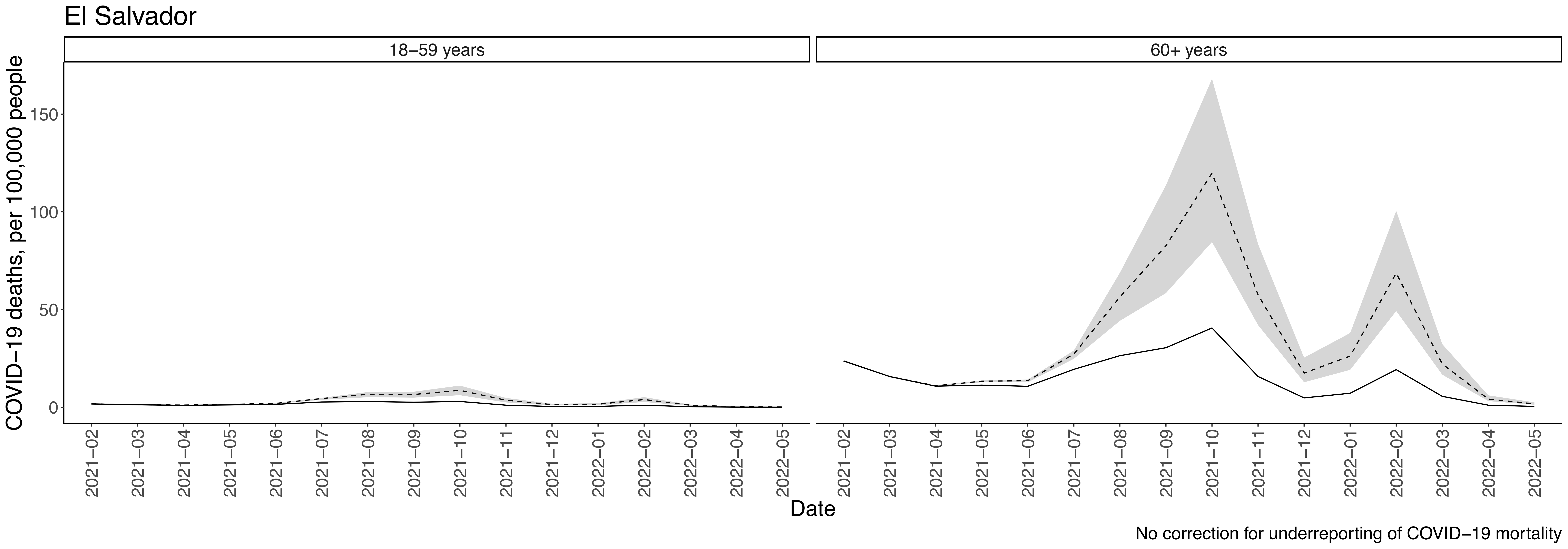
El Salvador
18–59 years
60+ years
COVID−19 deaths, per 100,000 people
0
50
100
150
2021−02
2021−03
2021−04
2021−05
2021−06
2021−07
2021−08
2021−09
2021−10
2021−11
2021−12
2022−01
2022−02
2022−03
2022−04
2022−05
Date
No correction for underreporting of COVID−19 mortality

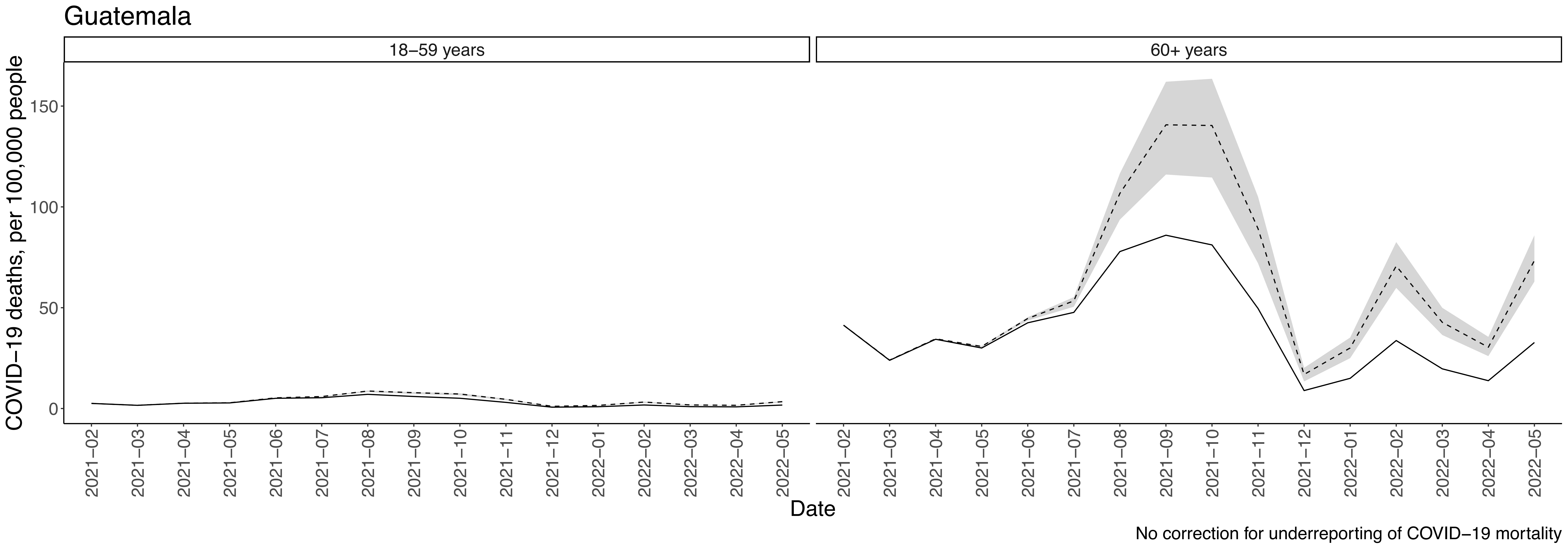
Guatemala
18–59 years
60+ years
COVID−19 deaths, per 100,000 people
0
50
100
150
2021−02
2021−03
2021−04
2021−05
2021−06
2021−07
2021−08
2021−09
2021−10
2021−11
2021−12
2022−01
2022−02
2022−03
2022−04
2022−05
Date
No correction for underreporting of COVID−19 mortality

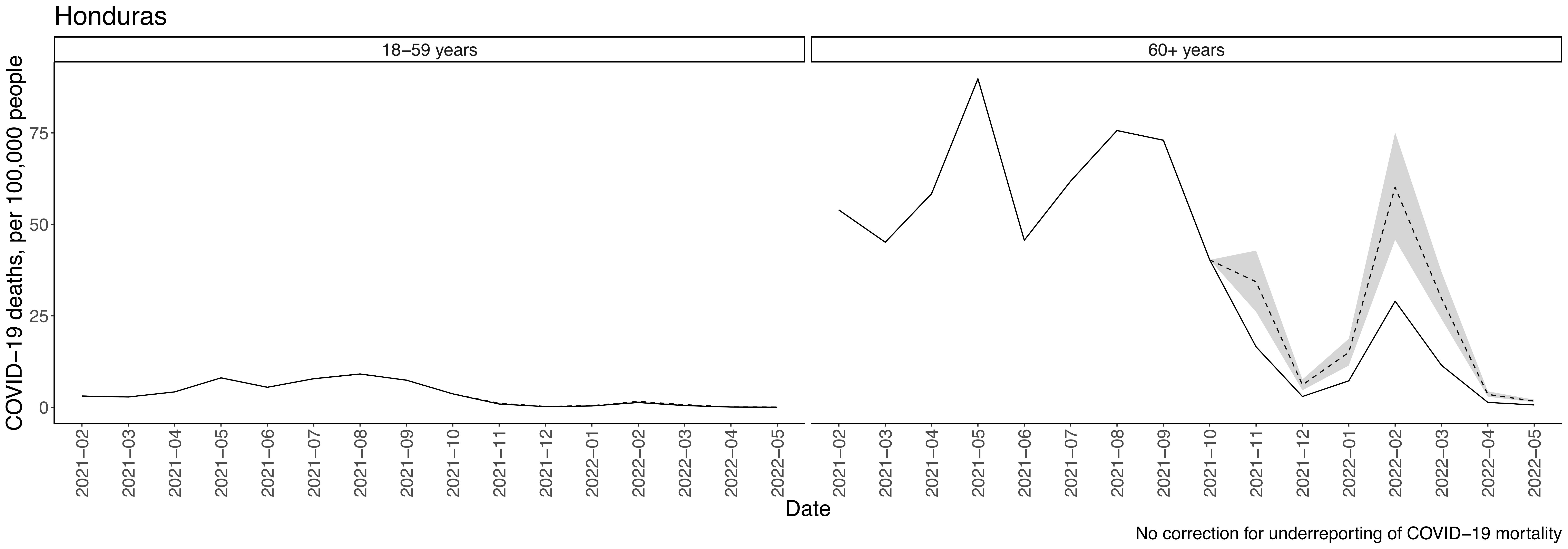
Honduras
18–59 years
60+ years
COVID−19 deaths, per 100,000 people
0
25
50
75
2021−02
2021−03
2021−04
2021−05
2021−06
2021−07
2021−08
2021−09
2021−10
2021−11
2021−12
2022−01
2022−02
2022−03
2022−04
2022−05
Date
No correction for underreporting of COVID−19 mortality

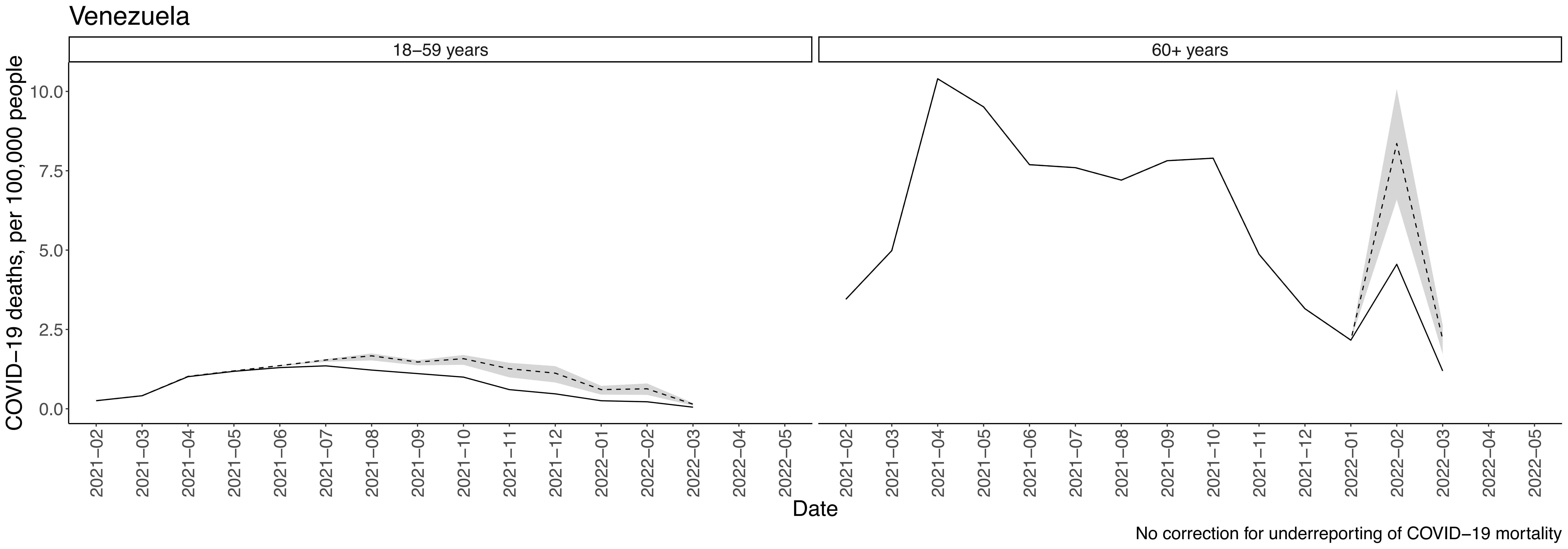
Venezuela
18–59 years
60+ years
COVID−19 deaths, per 100,000 people
10.0
7.5
5.0
2.5
0.0
2021−02
2021−03
2021−04
2021−05
2021−06
2021−07
2021−08
2021−09
2021−10
2021−11
2021−12
2022−01
2022−02
2022−03
2022−04
2022−05
Date
No correction for underreporting of COVID−19 mortality

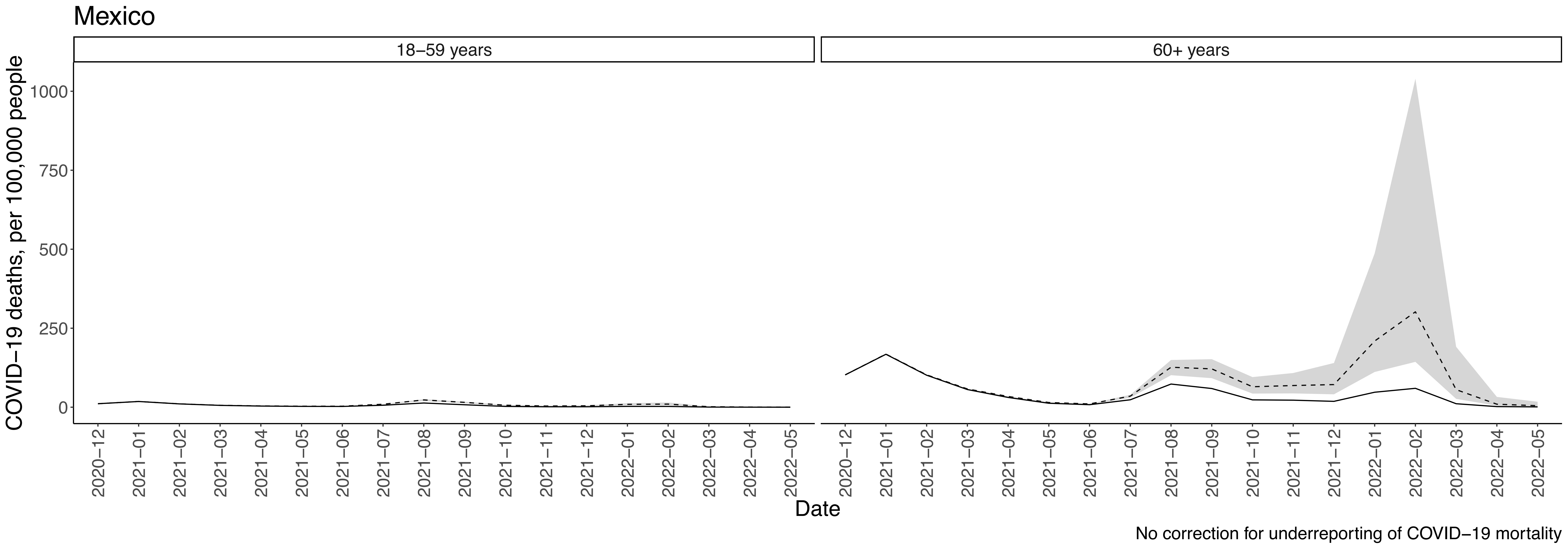
Mexico
18–59 years
60+ years
COVID−19 deaths, per 100,000 people
1000
750
500
250
0
2020−12
2021−01
2021−02
2021−03
2021−04
2021−05
2021−06
2021−07
2021−08
2021−09
2021−10
2021−11
2021−12
2022−01
2022−02
2022−03
2022−04
2022−05
Date
No correction for underreporting of COVID−19 mortality

Supplementary Figure 4.

Incident deaths averted per 100,000 population (vertical axis) plotted against vaccination coverage (two dose vaccination) (horizontal axis). A. Under 60 years of age, B. 60+ years of age.

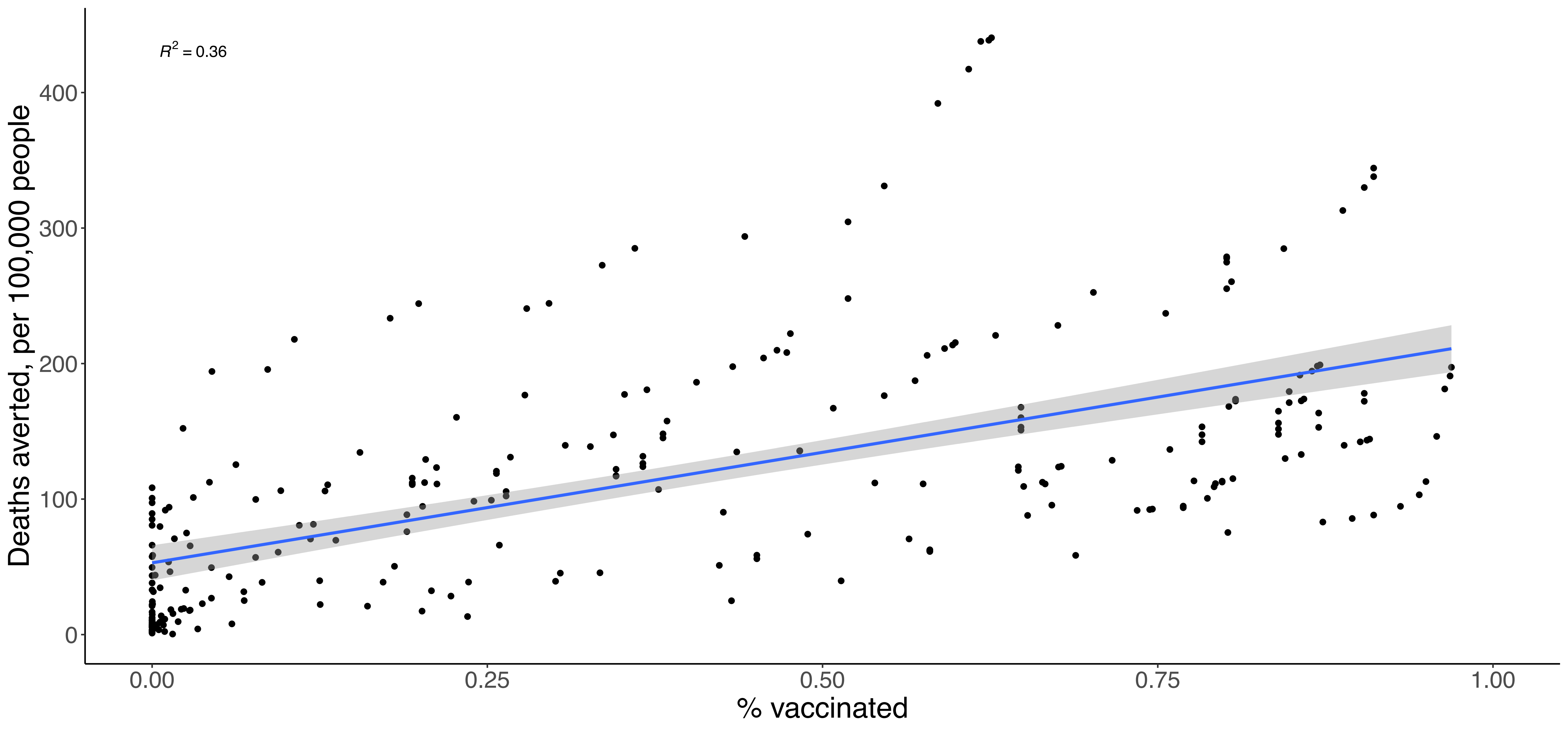

$R^2 = 0.36$
Deaths averted, per 100,000 people
400
300
200
100
0
0.00
0.25
0.50
0.75
1.00
% vaccinated

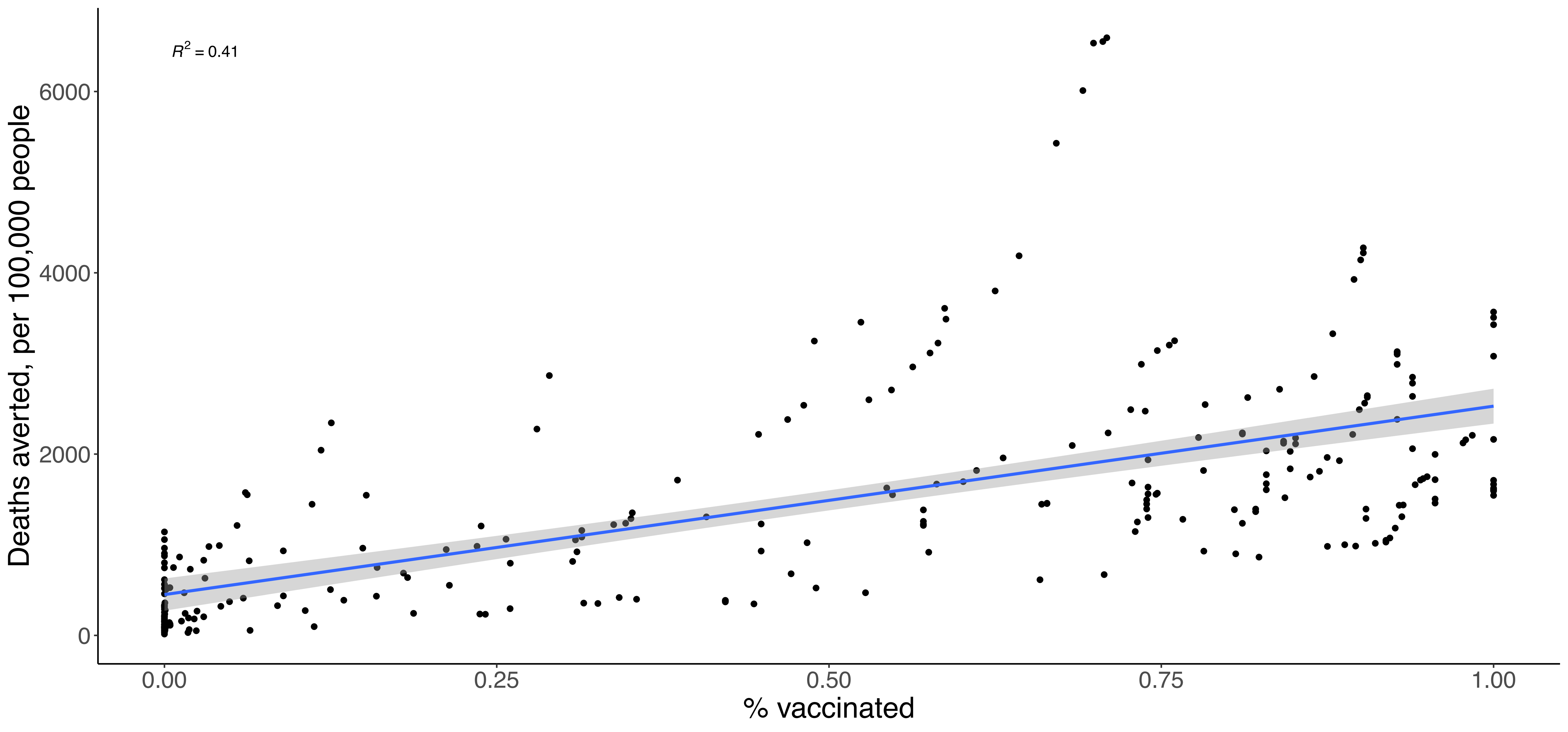

$R^2 = 0.41$
Deaths averted, per 100,000 people
6000
4000
2000
0
0.00
0.25
0.50
0.75
1.00
% vaccinated

Additional methods

Deaths by age imputation

COVID-19 death data by age (18-59, 60+) from the COVerAGE-DB database was unavailable for the following countries: Belize, Bolivia, Costa Rica, Ecuador, El Salvador, Guatemala, Honduras, and Venezuela. Mexico had partial age-specific mortality data but was missing age-specific vaccination data, and therefore Mexico data were not included when building the regression model and imputation was done for age-specific deaths in Mexico from November 2021 to May 2022. Deaths data for the time period in question was available from the WHO, however these data were not age-stratified. We therefore imputed proportion of deaths by age group for these countries using a linear regression, building the prediction model using data from countries with complete age-stratified COVID-19 deaths data from COVerAGE-DB.

Predictors used in the model were month and year as a categorical variable, proportion of population under 60 years of age, and the proportion of the population 60 and over who are vaccinated (partly and fully). Outcome predicted was proportion of deaths in those 60 and over. The prediction model was validated by comparing generated predicted values for countries with full data to observed proportions of death in those 60 and over. See Table below for observed and predicted values for each country.

	Argentina		Brazil		Chile		Colombia		Paraguay		Uruguay		Jamaica		Peru	
Month	Observed	Predicted	Observed	Predicted	Observed	Predicted	Observed	Predicted	Observed	Predicted	Observed	Predicted	Observed	Predicted	Observed	Predicted
Jan-21	0.14	0.17	0.19	0.20	0.14	0.17	0.18	0.22	0.31	0.24	-	0.09	0.21	0.26	0.28	0.21
Feb-21	0.13	0.20	0.23	0.22	0.14	0.17	0.19	0.25	0.34	0.27	-	0.11	0.27	0.28	0.31	0.23
Mar-21	0.15	0.22	0.28	0.24	0.17	0.17	0.20	0.27	0.32	0.30	-	0.05	0.31	0.30	0.32	0.26
Apr-21	0.18	0.23	0.32	0.25	0.24	0.18	0.25	0.28	0.34	0.33	0.14	0.05	0.19	0.34	0.32	0.28
May-21	0.26	0.27	0.40	0.28	0.26	0.22	0.32	0.30	0.35	0.38	0.19	0.04	0.17	0.37	0.30	0.33
Jun-21	0.33	0.31	0.50	0.33	0.22	0.29	0.40	0.34	0.32	0.44	0.21	0.11	0.34	0.41	0.36	0.38
Jul-21	0.31	0.31	0.44	0.33	0.20	0.31	0.37	0.32	0.36	0.40	0.22	0.15	0.26	0.42	0.47	0.36
Aug-21	0.26	0.27	0.31	0.30	0.16	0.29	0.29	0.28	0.33	0.38	0.27	0.14	0.36	0.33	0.40	0.32
Sep-21	0.23	0.19	0.23	0.24	0.15	0.23	0.23	0.27	0.32	0.31	0.18	0.14	0.30	0.28	0.32	0.26
Oct-21	0.22	0.17	0.19	0.22	0.17	0.19	0.20	0.24	0.37	0.27	0.10	0.11	0.26	0.26	0.24	0.24
Nov-21	0.20	0.18	0.21	0.22	0.15	0.20	0.17	0.30	0.34	0.28	0.13	0.11	0.32	0.26	0.21	0.25
Dec-21	0.16	0.22	0.24	0.26	0.14	0.24	0.18	0.25	0.35	0.32	-	0.15	0.62	0.26	0.23	0.28
Jan-22	0.12	0.17	0.17	0.21	0.14	0.18	0.11	0.22	-	0.27	-	0.09	0.51	0.21	0.20	0.22
Feb-22	0.13	0.14	0.15	0.18	0.09	0.16	0.09	0.19	-	0.24	-	0.07	0.48	0.19	0.17	0.20
Mar-22	0.15	0.11	0.16	0.16	0.10	0.13	0.12	0.24	-	0.22	-	0.05	0.23	0.21	0.24	0.17
Apr-22	0.22	0.15	0.21	0.20	0.10	0.19	0.16	0.22	-	0.27	-	0.10	-	0.25	0.27	0.21
May-22	0.14	0.14	0.17	0.18	0.13	0.15	0.11	0.22	-	0.24	-	0.07	-	0.23	0.29	0.17

Predicted values for proportion of deaths in those over 60 for countries with no data, as well as for countries with missing data (Paraguay, Uruguay, Jamaica) were then applied to the number of deaths observed in that month in order to divide deaths by age group.

National Underreporting Estimates

National underreporting estimates are taken from Msemburi et. al 2023.[1] To get an underreporting estimate, we compared reported number of COVID-19 deaths in 2021 to the number of excess deaths in the country during the same time frame. If the number of COVID-19 deaths exceeded excess deaths, we assumed there was no underreporting.

Country	ISO3	Year	Reported COVID Deaths	Excess Deaths	Underreporting
Antigua and Barbuda	ATG	2021	113	27.07	-3.1743628
Argentina	ARG	2021	74093	58190.5	-0.2732834
Bahamas	BHS	2021	546	667.44	0.18194894
Barbados	BRB	2021	253	-95.95	3.63679
Belize	BLZ	2021	356	574.98	0.38084803
Bolivia (Plurinational State of)	BOL	2021	10515	51731.99	0.79674086
Brazil	BRA	2021	426136	470456.44	0.09420732
Chile	CHL	2021	22597	24119.93	0.0631399
Colombia	COL	2021	87246	110683.86	0.21175499
Costa Rica	CRI	2021	5198	8290.03	0.37298176
Cuba	CUB	2021	8177	18393.56	0.55544223
Dominica	DMA	2021	45	69.64	0.35381964
Dominican Republic	DOM	2021	1837	10797.87	0.82987386
Ecuador	ECU	2021	19646	34465.67	0.42998352
El Salvador	SLV	2021	2496	9529.02	0.73806331
Grenada	GRD	2021	199	-13.68	15.5467836
Guatemala	GTM	2021	11299	40647.14	0.72202226
Guyana	GUY	2021	887	2415.5	0.63278824
Haiti	HTI	2021	530	6743.71	0.92140825
Honduras	HND	2021	7323	15377.4	0.52378165
Jamaica	JAM	2021	2168	4494.59	0.51764232
Mexico	MEX	2021	155119	311676.27	0.50230731
Nicaragua	NIC	2021	52	2928.99	0.98224644
Panama	PAN	2021	3492	4645.13	0.24824494

Paraguay	PRY	2021	14404	17406.87	0.17251062
Peru	PER	2021	109518	155851.18	0.29729117
Saint Kitts and Nevis	KNA	2021	28	-76.35	1.36673216
Saint Lucia	LCA	2021	290	479.91	0.39572003
Saint Vincent and the Grenadines	VCT	2021	83	386.55	0.78528004
Suriname	SUR	2021	1069	1013.26	-0.0550106
Trinidad and Tobago	TTO	2021	2699	1938.91	-0.3920192
Uruguay	URY	2021	6000	5351.6	-0.12116
Venezuela (Bolivarian Republic of)	VEN	2021	4300	18683.91	0.76985545

1. Msemburi W, Karlinsky A, Knutson V, Aleshin-Guendel S, Chatterji S, Wakefield J. The WHO estimates of excess mortality associated with the COVID-19 pandemic. *Nature* 2023; **613**(7942): 130-7.

Supplementary Table 1. Sensitivity analysis with observed and averted COVID-19 deaths via vaccination, by country, considering varying estimates of vaccine effectiveness, and adjustment for mortality under-reporting.

	**With adjustment for under-reporting (country-specific)**				**No adjustment for under-reporting**			
**Country**	Observed deaths, per 100,000 population	Deaths averted, medium vaccine effectiveness, per 100,000 population	Deaths averted, low vaccine effectiveness, per 100,000 population	Deaths averted, high vaccine effectiveness, per 100,000 population	Observed deaths, per 100,000 population	Deaths averted, medium vaccine effectiveness, per 100,000 population	Deaths averted, low vaccine effectiveness, per 100,000 population	Deaths averted, high vaccine effectiveness, per 100,000 population
**Argentina**	264.9	237.98	118.68	508.77	264.9	108.56	50.91	298.39
**Brazil**	309.0	250.15	123.83	589.95	279.9	98.47	43.02	251.02
**Chile**	216.5	588.29	395.26	1133.09	202.8	354.42	235.18	866.29
**Colombia**	301.9	207.48	110.58	330.17	238	74.78	28.17	150.87
**Paraguay**	424.4	220.53	136.41	311.47	351.2	104.58	56.84	188.70
**Uruguay**	252.1	795.24	485.55	4507.62	252.1	462.61	259.98	2133.38
**Jamaica**	250.1	47.27	33.09	61.45	120.6	14.18	4.73	23.63
**Peru**	656.9	372.24	226.21	651.75	461.6	144.30	84.50	351.01
**Belize**	230.8	75.82	37.91	113.74	142.9	37.91	0.00	37.91
**Bolivia**	757.8	428.42	262.84	595.33	154	49.94	27.60	91.99
**Costa Rica**	242.4	345.76	203.84	549.60	152	116.11	61.93	283.83
**Ecuador**	298.5	294.74	147.37	511.70	170.1	81.05	42.57	207.96
**El Salvador**	221.2	280.67	165.64	427.91	57.9	36.81	20.71	87.42
**Guatemala**	421.1	183.54	117.05	241.60	117	30.90	16.86	51.50
**Honduras**	236.8	20.02	12.32	27.72	112.8	4.62	1.54	9.24
**Venezuela**	105.9	18.24	10.73	24.14	24.4	2.68	2.15	5.36
**Mexico**	433.0	331.64	149.50	837.39	215.5	63.23	28.79	175.36
